# Adipose Tissue Engineering Biomaterials: Smart Scaffolds, Vascularization, and Clinical Frontiers

**DOI:** 10.3390/biom16030362

**Published:** 2026-02-28

**Authors:** Xin-Yi Zhao, Peng-Cheng Li, Yong-Mei Chen, Kai Cao, Wei Wei, Yasir Aziz, Miklós Zrínyi

**Affiliations:** 1 College of Bioresources Chemical and Materials Engineering, National Demonstration Center for Experimental Light Chemistry Engineering Education, Shaanxi University of Science and Technology, Xi’an 710021, China; 2 Second Affiliated Hospital of Xi’an Jiaotong University, Xi’an 710004, China; 3 Laboratory of Nanochemistry, Department of Biophysics and Radiation Biology, Semmelweis University, H-1089 Budapest, Hungary

**Keywords:** adipose tissue engineering, scaffold materials, adipogenic differentiation, angiogenesis, seed cells, growth factors

## Abstract

Adipose tissue engineering (ATE) is an interdisciplinary field integrating materials science, cell biology, and engineering, aiming to construct functional artificial adipose tissue for addressing adipose tissue deficiency, metabolic disorders, and related clinical challenges. This review systematically summarizes the core advances, critical limitations, and translational potential of ATE. First, we elaborate on the three fundamental elements of ATE: scaffold materials (hydrogels, porous materials, microspheres, fibrous materials, decellularized extracellular matrix, 3D-printed/bioprinted scaffolds, and prevascularized constructs), seed cells (adipose-derived stem cells, mesenchymal stem cells, etc.), and growth factors (vascular endothelial growth factor, fibroblast growth factor, etc.), as well as their synergistic regulatory roles in adipose tissue regeneration. We then discuss the key factors influencing adipogenic differentiation and vascularization, which are pivotal for the formation of functional ATE constructs. Furthermore, we detail the construction and evaluation of in vitro and in vivo ATE models, highlighting the value of large animal models in bridging preclinical and clinical gaps. The applications of ATE in soft tissue repair and reconstruction, drug screening and disease modeling, and cultured meat manufacturing are comprehensively analyzed, with emphasis on technical challenge across different directions. Finally, we discuss the core challenges hindering ATE clinical translation, including lack of standardization of adipose-derived stem cells, immunogenicity issues, regulatory barriers, and technical limitations, and propose targeted future perspectives. This review provides a comprehensive and critical overview of ATE, offering guidance for promoting its translation from preclinical research to clinical practice and industrial application.

## 1. Introduction

**Figure 1:**
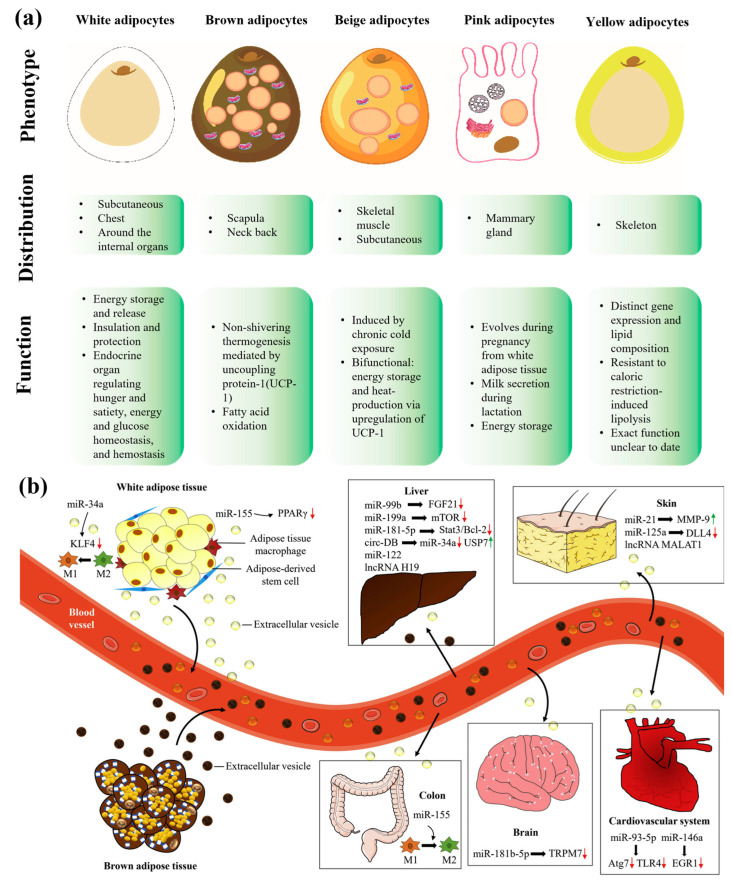
Classification and functions of adipocytes. (**a**) The schematic illustrations of adipocyte colors encompass white, brown, beige, pink, and yellow, and characteristic phenotypes, distribution and unique functions associated with each type of adipose tissue. Reproduced with permission from ref. [4]. Copyright 2020 Springer Nature. (**b**) The schematic illustration of adipose-derived extracellular vesicles enhances different organ crosstalk through blood circulation. Reproduced with permission from ref. [11]. Copyright 2021 Elsevier.

Adipose tissue is a specialized connective tissue primarily composed of adipocytes, which aggregate into distinctive lobular structures encased within fibrous networks. Adjacent lobules are separated by fibrous septa, and adipocytes are densely surrounded by capillary networks that supply essential nutrients and oxygen [1]. Based on structural and functional characteristics, mammalian adipose tissue is generally classified into white adipose tissue and brown adipose tissue [2]. Additional subtypes, including beige [3], yellow [4] and pink adipocytes, have also been identified [5,6] (Figure 1a). In humans, adipose tissue mass varies considerably among individuals, typically accounting for 15–25% of total body weight in men and 30–40% in women [7]. It plays indispensable roles in maintaining organismal homeostasis, including organ protection, energy storage, thermoregulation, metabolic regulation, and immune modulation [8,9,10]. Emerging evidence indicates that RNA components contained in adipose-derived extracellular vesicles exert therapeutic effects on distal organs such as the liver, skin, colon, brain, and heart. Adipose-derived extracellular vesicles secrete diverse functional RNAs, and their downstream regulatory targets in organ crosstalk are illustrated in Figure 1b [11,12].

While adipose tissue is essential for physiological homeostasis, excessive expansion leads to obesity, a major risk factor for hyperglycemia, hyperlipidemia, hypertension, and cardiovascular and cerebrovascular diseases [13]. Conversely, trauma, disease, or congenital defects that cause adipose tissue loss or dysfunction severely compromise physical and mental health [4,11]. Both excess and deficiency of adipose tissue represent pressing clinical challenges. A deeper understanding of the mechanisms governing adipose tissue growth, differentiation, and proliferation will facilitate improved management of obesity, reduce the incidence of metabolic comorbidities, and support the development of novel strategies for soft tissue repair. Traditionally, obesity-related mechanisms have been investigated using isolated cell cultures and animal models. However, conventional two-dimensional (2D) cell culture fails to recapitulate the three-dimensional (3D) microenvironment of native tissues. Although animal models partially mimic in vivo physiology, their high cost, long experimental cycles, and interspecies differences limit their translational value.

Tissue engineering provides a powerful and versatile platform to overcome these limitations. By directing cell cultivation, proliferation, and differentiation, tissue engineering enables the de novo formation of functional tissues, supporting investigations into lipid metabolism, obesity pathogenesis, drug screening, and tissue regeneration [14]. Furthermore, by precisely regulating the in vitro culture microenvironment, tissue engineering enhances adipocyte proliferation and differentiation. Combined with vascularization strategies, it improves vascular remodeling in implanted adipose constructs, reduces graft resorption, and enhances long-term retention [15]. These advances directly address the critical clinical challenge of graft atrophy in autologous fat transplantation, offering transformative potential for reconstructive surgery and regenerative medicine.

Tissue engineering scaffolds are commonly categorized into natural and synthetic biomaterials [16]. Synthetic biomaterials exhibit favorable structural stability but often suffer from suboptimal biocompatibility and biodegradability, introducing uncertainties and risks in clinical applications. Autologous fat grafts are attractive due to excellent biocompatibility and low immunogenicity. In comparison, natural biomaterials including polysaccharides (e.g., chitosan, alginate, hyaluronic acid), proteins (e.g., collagen, silk fibroin, gelatin), and decellularized tissues possess outstanding biocompatibility. In adipose tissue engineering, natural biomaterials offer core advantages through biomimetic architecture and multi-dimensional functional regulation. By preserving native extracellular matrix (ECM) components, they establish an in vivo-like 3D microenvironment that markedly promotes the adhesion, proliferation, and adipogenic differentiation of adipose-derived stem cells (ADSCs) [17]. Their low immunogenicity, derived from decellularization or autologous origin, minimizes immune rejection and enables personalized transplantation [16]. Synergistic effects between porous structures and bioactive factors (e.g., VEGF) accelerate vascular network formation, thereby improving graft survival [18,19]. Meanwhile, the controllable degradability of materials such as collagen and silk fibroin dynamically matches tissue regeneration, preventing long-term foreign-body retention [20,21]. Integrated with 3D bioprinting, natural biomaterials permit the precise fabrication of biomimetic adipose constructs. By regulating lipid droplet formation and paracrine signaling, these materials enhance adipogenesis and vascularization, providing safe and effective tissue-engineering solutions for clinical soft tissue defect repair.

**Figure 2:**
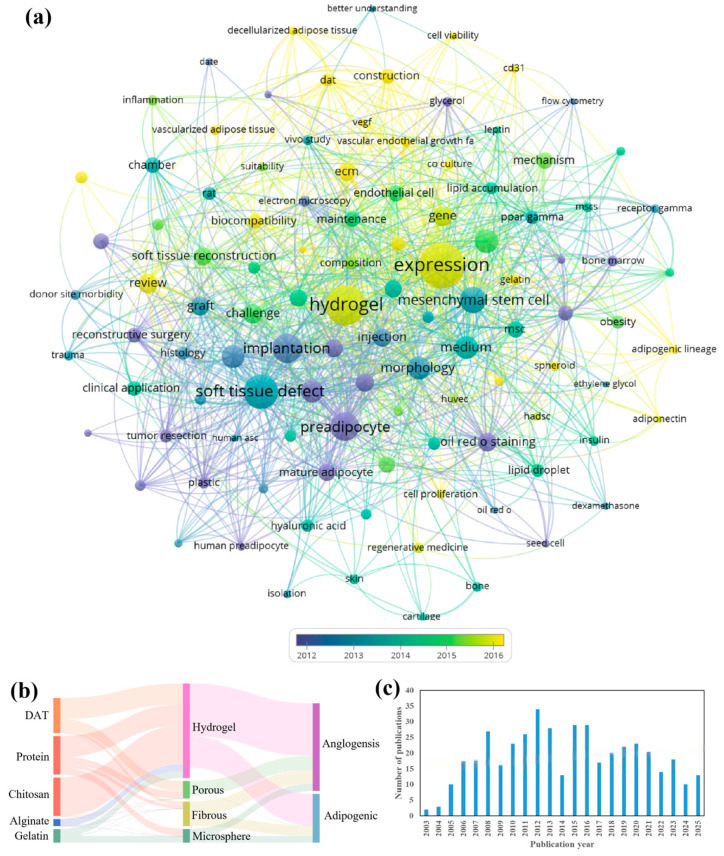
The research trends within disciplines where “adipose tissue engineering or tissue engineering adipose” as pivotal keyword. (**a**) Cluster analysis of recent research hotspots. (**b**) Visualization of different biomass materials and their use in adipose tissue engineering. (**c**) Number of publications per year from 2003 to 2025 via Web of Science.

Tissue engineering has become a central discipline in adipose tissue pathology and defect repair, promising more effective and durable clinical interventions. Persistent challenges in adipose tissue engineering (ATE) including maintaining cellular stability, preserving functional integrity, and optimizing biomaterial biocompatibility are gradually being resolved through scientific and technological innovation, highlighting a rapidly evolving field with enormous translational potential. ATE is expected to drive breakthroughs in the treatment of lipid metabolism disorders, soft tissue reconstruction, and even cultured meat production. To visualize the global research landscape, we performed a bibliometric analysis using the Web of Science database with the keywords “adipose tissue engineering OR tissue engineering adipose” from 2004 to 2026, retrieving 439 articles analyzed by VOSviewer (Figure 2). The specific operational details, as follows:

First, the original literature data were exported as a tab-delimited file with complete literature records and cited references included. The file was then imported into VOSviewer software (2025) for bibliometric analysis. For the Type of analysis, co-occurrence was selected, and the screening criterion of Minimum number of occurrences of a keyword was set to 5 to perform keyword co-occurrence cluster analysis on the literature set. After iterative calculation and clustering of the software based on the co-occurrence frequency and correlation of keywords, the final cluster analysis results were obtained (Figure 2a).

Subsequently, we conducted multi-dimensional classification of the screened literature according to three core dimensions. The first dimension was the type of biomass materials, including alginate, chitosan, DAT, gelatin, and protein. The second dimension was the structural type of scaffolds prepared from the above materials, including fibrous, hydrogel, microsphere, and sponge scaffolds. The third dimension was the functional classification of the scaffolds, limited to Angiogenesis and adipogenic differentiation, the two core functional characteristics focused on in this study. Based on the above three-dimensional classification results, we collated the corresponding quantitative association data between each classification level, and used the online Sankey diagram visualization tool (https://www.lddgo.net/chart/sankey-chart, accessed on 26 September 2025) to draw the Sankey diagram. The data input followed the tool’s standard format (i.e., the first column as the source node, the second column as the target node, and the third column as the quantitative value of the association between nodes), and the diagram was optimized by setting parameters such as horizontal display direction, left-right justified alignment, and bright theme to clearly visualize the quantitative association relationships among biomass material types, scaffold structural types and scaffold functional characteristics (Figure 2b).

Although previous reviews have summarized biomaterials for adipose vascularization, few have systematically elucidated how scaffold biomaterials regulate stem cell adipogenic differentiation and angiogenesis. Moreover, the influence of scaffold morphology on adipose vascularization remains underexplored, and standardized strategies for ATE model construction and evaluation are lacking.

In this review, we comprehensively summarize recent advances in adipose tissue engineering, with a special emphasis on biomaterials. We first introduce the three core components of ATE (seed cells, growth factors, and scaffolds), and discuss their respective functions. We then highlight the molecular mechanisms underlying stem cell adipogenic differentiation and angiogenesis, emphasizing the pivotal role of vascularization in ATE. We further analyze how different categories of biomaterial scaffolds modulate adipose tissue vascularization and outline key standards for ATE model establishment and characterization. We also review the expanding applications of ATE in the comprehensive health industry to promote its high-quality development. Finally, we discuss current challenges and propose future perspectives to guide the next wave of innovation in adipose tissue engineering.

## 2. Key Elements of ATE

As a specialized branch of tissue engineering, adipose tissue engineering (ATE) integrates insights and knowledge from materials science, cell biology, engineering, and surgery, with the core goal of constructing functional artificial adipose tissue to restore the normal structure and functionality of damaged adipose tissue. The core process of ATE involves first obtaining adipose-derived tissue fragments or target seed cells (e.g., adipose-derived stem cells) from living organisms, which are then cultured and expanded in vitro to ensure sufficient cell quantity and viability. Subsequently, the expanded cells are seeded onto a biomaterial scaffold that mimics the natural adipose tissue microenvironment, where they undergo migration, growth, proliferation, and adipogenic differentiation to form a cell-scaffold composite with distinct morphological and functional characteristics similar to natural adipose tissue [22]. This composite is then implanted into the adipose lesion site to regenerate damaged adipose tissue, ideally restoring its original structure, volume, and physiological functions (e.g., lipid storage, metabolic regulation) and ultimately achieving the goals of adipose tissue repair, defect reconstruction, and functional recovery [23,24].

The essence of ATE lies in constructing a 3D structure that highly mimics natural adipose tissue, which is composed of biocompatible scaffolds and multiple adipose-related cell types. The successful implementation of ATE relies on three core and mutually coordinated elements: the selection and culture of adipose-specific seed cells, the design and preparation of bioactive scaffolds suitable for adipogenic growth, and the precise regulation of adipose tissue structure and function reconstruction. Through the synergistic regulation of these three core elements, biologically active, structurally stable, and functionally viable artificial adipose tissue can be successfully constructed, laying a foundation for clinical application in adipose tissue defect repair, soft tissue augmentation, and related disease treatment.

### 2.1. Scaffold Materials for ATE

Scaffold materials are the core structural foundation of ATE, serving as the “supporting framework” for adipose-related seed cell growth, proliferation, and adipogenic differentiation. The biomaterials of scaffolds for ATE construction in medicine are mainly classified into synthetic biomaterials, natural biomaterials, and decellularized adipose tissue (DAT). The synthetic biomaterials include polylactic acid (PLA), polyglycolic acid (PGA), polylactic-co-glycolic acid (PLGA), and aliphatic polyesters, etc. While these synthetic materials possess biocompatibility and can be tailored and designed on demand to fabricate scaffolds that meet target requirements, their performance in terms of biocompatibility, immune response, degradability, and matching degree with the growth rate of new tissues remains unsatisfactory. The natural biomaterials are mainly classified into polysaccharides (e.g., chitosan, alginate, hyaluronic acid, and its derivatives), proteins (e.g., collagen, fibrin, silk fibroin, etc.) and nucleotides (e.g., DNA and RNA). These biomaterials are usually highly biocompatible and provide a suitable environment for cells to survive. DAT, as a special type of biological scaffold, can effectively reconstruct the natural extracellular environment by removing cellular components while retaining abundant collagen and glycosaminoglycan matrices, thereby promoting the specific differentiation and renewal of stem cells, facilitating the reconstruction of vascular and neural networks, and providing a highly biocompatible and physiologically relevant microenvironment for ATE construction [23]. Based on their structural and functional properties, all the aforementioned scaffold materials can be further classified into hydrogels, porous materials, microspheres, and fibrous materials, which will be discussed in detail in Section 5.

### 2.2. Seed Cells for ATE

Seed cells are the core functional units of ATE, and their adipogenic differentiation potential, proliferation capacity, and biocompatibility directly affect the quality and functional stability of engineered adipose tissue. Seed cells commonly employed in ATE include various stem cells and adult cells. Stem cell types include embryonic stem cells (ESCs), mesenchymal stem cells (MSCs), adipose-derived stem cells (ADSCs), and Wharton’s jelly mesenchymal stem cells (WJCMSCs), while adult cells include human umbilical vein endothelial cells (HUVECs) and adipocytes. ESCs are characterized by their pluripotency, i.e., the ability to differentiate into any cell type in the human body; however, ethical controversies and the risk of immune rejection associated with ESCs have shifted research focus toward alternative stem cell sources [25].

MSCs have attracted considerable attention due to their multipotent differentiation potential and ease of isolation from various tissues, such as adipose tissue, bone marrow, immature teeth, and umbilical cord blood. Additionally, MSCs have the advantage of reducing the risk of immune responses, potentially avoiding complications associated with allogeneic transplantation [26]. Among these stem cell types, ADSCs have emerged as a particularly attractive candidate for ATE, primarily due to their abundance and non-invasive accessibility via liposuction. ADSCs also exhibit remarkable multipotent differentiation capacity, including the ability to differentiate into adipocytes, further confirming their suitability for ATE applications [27]. WJCMSCs, as a rich source of pluripotent cells, have demonstrated great potential in ATE. The non-invasive collection process (which causes no harm to the donor) and their high expansion rate make WJCMSCs a valuable supplementary seed cell resource for ATE, especially when ADSCs are difficult to obtain [28].

In scenarios where immune compatibility is critical, mature adipocytes can serve as direct seed cells in tissue engineering. HUVECs are also widely used due to their strong capacity to form vascular networks, a key prerequisite for the survival and function of engineered adipose tissue. These cells supply nutrients and oxygen to the engineered construct while removing metabolic waste, thereby maintaining the viability and functionality of the engineered tissue [29]. Notably, adipocytes derived from the patient’s own adipose tissue can bypass immune rejection complexities, facilitating more harmonious integration into the host tissue environment [30].

Current research and clinical applications of ATE mainly focus on ADSCs, MSCs, and HUVECs. These cell types are favored for their combined advantages of abundant sources, high proliferative capacity, and low immunogenicity. Specifically, these cells can be induced to differentiate into adipocytes and endothelial cells, which serve as the foundation for the formation of adipose tissue and vascular networks, respectively [31,32,33,34].

### 2.3. Growth Factors

Growth factors are indispensable regulatory factors in ATE, as a family of highly bioactive peptides that play crucial roles in regulating the growth, proliferation, adipogenic differentiation of adipose-related seed cells, and the formation of adipose tissue and its vascular networks. In ATE research and application, vascular endothelial growth factor (VEGF), fibroblast growth factor (FGF), insulin-like growth factor (IGF), platelet-derived growth factor (PDGF), epidermal growth factor (EGF), and bone morphogenetic protein 4 (BMP4) are the most widely used types, each playing a unique and synergistic role in ATE. The biological activity and mechanism of action of these growth factors are closely related to the physiological process of adipose tissue formation and repair, and their rational application is crucial for optimizing ATE strategies.

In ATE, VEGF is the core growth factor regulating angiogenesis. It mainly binds specifically to VEGFR receptors on the surface of vascular endothelial cells, activates signaling pathways such as PI3K/Akt, promotes the proliferation, migration, and tube formation of human umbilical vein endothelial cells (HUVECs), and then helps to construct a complete vascular network, solving the problem of graft absorption caused by hypoxia and insufficient nutrient supply after engineered adipose tissue transplantation [35,36]. Li et al. have shown that combining VEGF sustained-release systems with adipose tissue engineering scaffolds can significantly increase the vascular density in the scaffold, promote the differentiation of ADSCs into mature adipocytes, and improve the long-term retention rate of grafts [37]. In addition, VEGF can also regulate the adipogenic differentiation potential of ADSCs through paracrine action, and cooperate with adipogenic induction factors to accelerate the maturation of artificial adipose tissue [38].

The FGF family mainly undertakes dual regulatory roles in ATE, participating in both adipogenic differentiation of adipocytes and auxiliary vascular network construction. Among them, basic fibroblast growth factor (bFGF) is the most widely used. It can down-regulate the expression of adipogenic inhibitors by activating the ERK1/2 signaling pathway, promote the adipogenic differentiation of ADSCs and MSCs, and induce the proliferation of vascular endothelial cells, cooperating with VEGF to enhance the vascularization efficiency of scaffolds [39]. Chen et al. have confirmed that loading bFGF on collagen scaffolds can significantly increase the lipid droplet accumulation of ADSCs, promote the formation of new blood vessels in the scaffold, and provide stable support for the growth of engineered adipose tissue [40]. In addition, FGF can also regulate the synthesis and remodeling of adipose tissue matrix, enhancing the structural stability of artificial adipose tissue [41].

IGF is a key adipogenic induction growth factor in ATE. After binding to insulin receptors, it activates the PI3K/Akt/mTOR signaling pathway, accelerates the differentiation of ADSCs into adipocytes, promotes the synthesis and accumulation of lipid droplets, inhibits the apoptosis of adipocytes, and maintains the functional stability of artificial adipose tissue [42,43]. Huang et al. have reported that treating ADSCs with IGF-1 combined with adipogenic induction medium can significantly improve the efficiency of adipogenic differentiation, shorten the differentiation cycle, and produce induced adipocytes that are more similar to natural adipocytes in morphology [44]. In addition, IGF can also indirectly promote the vascularization of engineered adipose tissue by regulating the proliferation and migration of vascular endothelial cells, and cooperate with VEGF to further improve the survival quality of grafts [45].

The core role of PDGF in ATE is to regulate the proliferation of stromal cells and tissue remodeling. It mainly acts on mesenchymal cells such as MSCs and fibroblasts, promotes the proliferation and migration of these cells by activating the PLCγ signaling pathway, and then promotes the synthesis of extracellular matrices such as collagen and glycosaminoglycan, enhances the adhesion between scaffold and cells, and provides structural support for angiogenesis [46,47]. Deng et al. have found that the combined application of PDGF and VEGF can significantly improve the vascularization level and matrix remodeling efficiency of engineered adipose tissue, reduce graft fibrosis, and enhance the biocompatibility and functional integrity of artificial adipose tissue [48]. In addition, PDGF can also reduce the risk of post-transplant immune rejection by inhibiting inflammatory responses, providing guarantee for the long-term survival of artificial adipose tissue [49].

Although EGF is relatively less used in ATE, it also plays an important auxiliary role. It mainly promotes the proliferation of epithelial cells, protects the integrity of the surface epithelium of engineered adipose tissue, reduces the risk of post-transplant infection, and can slightly promote the proliferation of ADSCs, providing sufficient cell sources for adipogenic differentiation [50,51].

BMP4 is an important adipose-related growth factor in ATE. Its core function is to mediate the interaction between adipogenic differentiation and angiogenesis, and to play a unique regulatory role in the maturation and functional stability of artificial adipose tissue [52,53]. BMP4 mainly activates the Smad1/5/8 signaling pathway. On the one hand, it directly promotes the differentiation of seed cells such as ADSCs and MSCs into mature adipocytes, accelerates the accumulation of lipid droplets, and regulates the morphogenesis of adipose tissue [54]. On the other hand, it can induce the proliferation and tube formation of vascular endothelial cells through paracrine mode, and up-regulate the expression of angiogenesis factors such as VEGF, realizing the coordinated regulation of adipogenic differentiation and angiogenesis, and solving the problem of “asynchrony between adipogenesis and vascularization” under single-factor regulation [55]. Wang et al. have confirmed that loading BMP4 and VEGF on biological scaffolds can significantly improve the adipogenic efficiency and vascular density of engineered adipose tissue, reduce graft fibrosis, and enhance the integration ability between artificial adipose tissue and host tissue [56]. In addition, BMP4 can also further optimize the scaffold microenvironment by regulating the synthesis of extracellular matrix, providing more suitable conditions for the growth and differentiation of seed cells [57].

In summary, growth factors such as VEGF, FGF, IGF, PDGF, and BMP4 have their own roles and synergistic effects in ATE. In-depth study of the specific mechanism of action, application mode, and synergistic effect of each growth factor in ATE not only provides a theoretical basis for optimizing ATE strategies but also lays a foundation for improving the clinical transformation efficiency of artificial adipose tissue [58]. At present, the application of growth factors in ATE still focuses on the optimization of sustained-release delivery systems (such as loading on scaffold materials to achieve long-term release), the synergistic ratio of multiple factors, and individualized application. Relevant studies provide new technical paths for solving key problems such as graft absorption and insufficient vascularization [59,60].

## 3. Effect Factors of Adipogenic Differentiation

**Figure 3:**
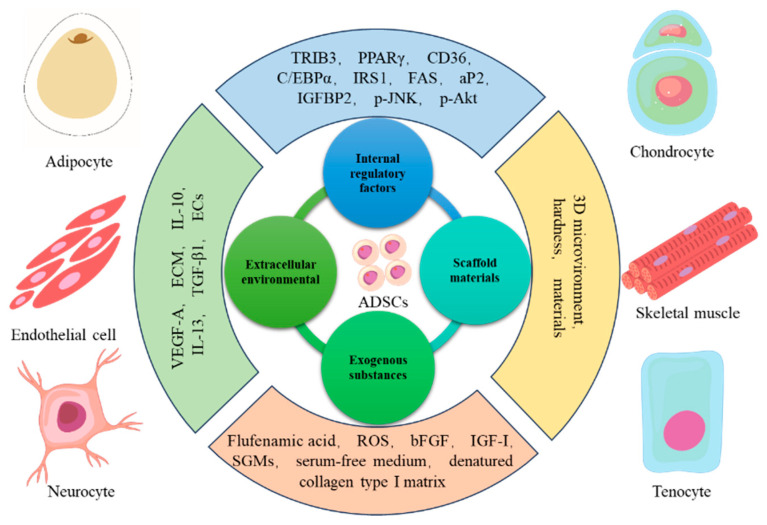
The factors that affect the differentiation of ADSCs in ATE include extracellular environmental, internal regulatory factors, scaffold materials, and exogenous substances. Schematic illustration of ADSCs have the ability to differentiate into adipocytes, endothelial cells, neurons, chondrocytes, skeletal muscle cells, and tenocytes.

Adipose-derived stem cells (ADSCs) possess self-renewal capacity, enabling them to generate at least one type of functionally highly differentiated daughter cell. The self-renewal potential of ADSCs varies depending on their specific subpopulation and the surrounding physiological microenvironment, ranging from short-term to long-term, or even infinite [61]. The adipogenic differentiation potential of ADSCs is precisely regulated by a complex network consisting of external signals and intrinsic gene expression, which guides ADSCs to differentiate into various cell types under specific developmental stages and environmental conditions (Figure 3) [62]. As a core seed cell for ATE, the adipogenic differentiation efficiency of ADSCs directly determines the success of artificial adipose tissue construction. Therefore, systematically exploring the factors influencing adipogenic differentiation is of great significance for optimizing ATE strategies and promoting its clinical transformation.

### 3.1. Internal Regulatory Factors

Internal regulatory factors, including specific genes, microRNAs (miRNAs), and cell subpopulations, play a decisive role in regulating the adipogenic differentiation of ADSCs. The ternary pseudokinase 3 (TRIB3) is a key negative regulatory factor in ADSC adipogenic differentiation. Lipid droplet formation is significantly increased in TRIB3 knockdown ADSCs, accompanied by the up-regulation of lipogenic genes such as peroxisome proliferator-activated receptor gamma (PPARγ), cluster of differentiation 36 (CD36), and CCAAT/enhancer binding protein alpha (C/EBPα), which collectively enhance the lipogenic capacity of ADSCs. In contrast, the overexpression of TRIB3 significantly down-regulates the expression of these lipogenic genes, thereby inhibiting adipogenic differentiation and lipid droplet formation in ADSCs [63].

MicroRNAs also play an important regulatory role in adipogenic differentiation. Although most miRNAs promote adipogenesis, some specific miRNAs exhibit an inhibitory effect. For example, overexpression of miR-145 in porcine preadipocytes inhibits the mRNA expression of lipogenic markers (C/EBPα and PPARγ2) by targeting insulin receptor substrate 1 (IRS1), thereby suppressing adipogenic differentiation [64]. In addition, key lipogenic genes such as PPARγ2, fatty acid synthase (FAS), adipocyte fatty acid-binding protein (aP2), and perilipin are closely involved in the dynamic process of adipogenic differentiation, regulating the initiation, progression, and maturation of adipogenesis [65].

The heterogeneity of ADSC subpopulations also leads to differences in their adipogenic differentiation potential. ADSCs exist in different anatomical locations, including superficial subcutaneous adipose tissue, deep subcutaneous adipose tissue, and superficial retinacula cutis, with distinct functional characteristics. For instance, deep subcutaneous adipose tissue has the strongest lipolytic capacity, superficial subcutaneous adipose tissue exhibits the highest adipogenic induction potential, and superficial retinal cells show the strongest ability to secrete endothelial growth factor [19]. ADSCs with different lipogenic differentiation potentials (CD29^+^, CD71^+^, CD73^+^, and CD90^+^) can be isolated from rat adipose tissue using magnetic activated cell sorting. Among them, CD29^+^ cells have the highest adipogenic differentiation potential, and CD71^+^, CD73^+^, and CD90^+^ cells have significantly lower corresponding potential [66]. Additionally, four distinct stromal populations can be isolated from adult adipose tissue, among which the CD31^−^/CD34^+^ cell population is the most abundant and possesses the greatest adipogenic differentiation potential, providing a reliable cell source for ATE applications [67].

In addition to ADSCs, other stem cells and mature adipocytes also exhibit varying adipogenic differentiation capacities that affect ATE outcomes. Mature adipocytes are potential seed cells for ATE. Fibroblast-like adipocytes derived from mature adipocytes have a certain degree of differentiation capacity, even exceeding that of ADSCs. This phenomenon suggests that mature adipocytes can acquire a more robust ability to contribute to adipogenesis under specific inductive conditions, thereby broadening our understanding of adipocyte plasticity and differentiation dynamics [68]. Periodontal ligament stem cells (PDLSCs) and Wharton’s jelly mesenchymal stem cells (WJCMSCs) are also recognized for their adipogenic differentiation capabilities. Notably, WJCMSCs have weaker adipogenic differentiation potential compared to PDLSCs. The up-regulation of insulin-like growth factor-binding protein 2 (IGFBP2) has been demonstrated to significantly enhance the adipogenic differentiation of WJCMSCs and BMSCs. This enhancement is associated with increased phosphorylation of key signaling molecules, specifically c-Jun N-terminal kinase (p-JNK) and protein kinase B (p-Akt). The activation of the JNK and Akt signaling pathways, in turn, has been linked to the promotion of adipogenic differentiation in these stem cell populations. These findings highlight the role of IGFBP2 in modulating stem cell fate towards adipogenesis, and suggest a potential therapeutic strategy for augmenting adipose tissue formation [69].

Moreover, the impact of passage number on the differentiation potential of ADSCs is a critical consideration. It has been observed that freshly isolated ADSCs demonstrate a superior capacity for differentiation when compared to cells that have been cultured for eight passages. This finding emphasizes the need for careful selection of cell passages in experimental design and result interpretation to ensure the efficiency of ATE [70].

### 3.2. Extracellular Environmental Factors and Intercellular Interactions

The adipogenic differentiation of ADSCs is not only regulated by intrinsic genetic programs but also significantly influenced by the extracellular microenvironment and intercellular interactions, which together form a complex regulatory network to modulate adipogenesis. The adipose tissue microenvironment, particularly the extracellular matrix (ECM) and secretory factors derived from adipose tissue, plays a crucial role in regulating ADSC behavior. Secretory factors from adipose tissue explants have been shown to suppress the proliferation and colony formation of ADSCs while simultaneously up-regulating the expression of adipogenic markers (including C/EBP, PPARγ2, lipoprotein lipase) and the angiogenic factor vascular endothelial growth factor A (VEGF-A), thereby promoting both adipogenesis and angiogenesis in vitro and in vivo [71]. Additionally, the vascular matrix fraction can promote the recruitment of host adipocytes, further enhancing adipose tissue generation [72].

The adipose ECM, which is devoid of cellular components but rich in collagen and 29 lipid metabolism-related proteins, plays an important regulatory role in adipogenic differentiation [73]. It can interact with various types of MSCs, regulate PPARγ expression, and promote fat regeneration, thereby providing a suitable microenvironment for adipocyte growth and differentiation. However, the purity of ECM preparation is critical. Impurities such as residual immunogenic antigens can trigger adverse immune responses. Therefore, it is imperative to adopt appropriate isolation methods to effectively remove immunogenic substances while retaining bioactive components in the ECM. For example, Liu, K., et al. [74] employed the methoxy polyethylene glycol (mPEG) decellularization technique to preserve bioactive factors and reduce immune reactions. In their study, adipogenesis induced by acellular adipose matrix transplantation was significantly lower in the wild-type group than in the immunodeficient group. After xenotransplantation, mPEG-modified acellular adipose matrix exhibited reduced immune responses and increased adipocyte formation compared to unmodified acellular adipose matrix. Furthermore, mPEG modification increased the number of regulatory T (Treg) cells in acellular adipose matrix grafts, which subsequently elevated the M2/M1 macrophage ratio by secreting IL-10, IL-13, and TGF-β1, potentially reducing the immunogenicity of xenograft acellular adipose matrix and promoting adipogenesis. In addition, co-culture of ADSCs with rat adipose ECM not only enhances angiogenesis and adipogenesis but also stimulates macrophage expression and induces inflammatory reactions, indicating the complex regulatory role of ECM in the adipose microenvironment [75]. Clinical studies have also shown that the adipogenesis rate in the experimental group treated with autologous adipose tissue is significantly higher than that in the control group, and the adiposity level is dependent on the amount of autograft [76]. Intercellular communication is another key factor regulating the proliferation and differentiation of stem cells in ATE. For example, the survival rate of preadipocytes decreases in hypoxic environments, but co-culture with endothelial cells can promote the release of soluble factors that maintain preadipocyte viability, although the specific characteristics of these factors require further investigation [77]. When adipocytes and endothelial cells are co-cultured on 3D porous fiber scaffolds, high concentrations of insulin stimulate triglyceride accumulation and reduce lipolysis, whereas hydrogels with different insulin concentrations have no significant effect on adipocyte monocultures [78]. This finding highlights the critical role of intercellular interactions in adipogenic differentiation, suggesting that the construction of multi-cell co-culture systems may be an effective strategy to optimize ATE.

### 3.3. Regulation of Exogenous Substances on Adipogenesis and Differentiation

The addition of exogenous substances is an effective strategy to regulate the adipogenic differentiation of ADSCs, with various compounds (e.g., flufenamic acid, bFGF, denatured type I collagen matrix) and culture conditions (e.g., serum-free medium) having been shown to modulate adipogenesis. Flufenamic acid directly regulates the expression of key adipogenic markers at both the gene and protein levels. It enhances the expression of PPARγ2 and perilipin, which play vital roles in the early and late stages of adipogenesis, thereby significantly promoting lipogenic differentiation [79]. Reactive oxygen species (ROS) also promote the adipogenic differentiation of ADSCs, and the combination of adipogenic induction medium and H_2_O_2_ exhibits a more significant promoting effect than either treatment alone [80].

Growth factors such as bFGF also play an important role in regulating adipogenic differentiation. Rats treated with gelatin-based microspheres immobilized with bFGF, insulin, and IGF-I showed significantly higher triglyceride levels at the injection site compared to those treated with microspheres immobilized only with insulin and IGF-I, indicating that bFGF can effectively promote adipose tissue neogenesis [81]. Culture conditions also affect the adipogenic potential of stem cells. Serum-free medium has been shown to be effective for ADSC expansion, improving cell proliferation and enhancing adipogenic potential [82]. Compared with traditional fetal bovine serum (FBS)-containing medium, serum-free medium increases the expression of PPARγ and lipid accumulation in human BMSCs (hBMSCs), thereby maintaining and enhancing their chondrogenic and adipogenic differentiation capacities. This finding highlights the importance of serum-free conditions in optimizing the differentiation potential of hBMSCs for regenerative medicine applications [83]. Additionally, the denatured type I collagen matrix can maintain the expression of adipocyte-associated markers (e.g., fatty acid-binding protein-4, lipoprotein lipase, acyl-CoA synthetase, adipsin, facilitative glucose transporter-4) and lipid accumulation in late-passage hBMSCs. This preservation of marker expression and function is crucial for the expansion of functional adipocytes, ensuring the production of viable adipogenic progenitors for ATE and regenerative medicine [84].

### 3.4. Effect of Scaffold Materials on Adipogenic Differentiation

In ATE, the selection of appropriate scaffold materials is crucial for regulating adipogenic differentiation. Scaffolds not only provide physical support for cells but also mimic the in vivo cellular growth environment, thereby influencing cell morphology, function, metabolism, and intercellular interactions. Ideal scaffolds should possess a 3D structure similar to native adipose tissue, appropriate stiffness, sufficient nutrient transport capacity, and the ability to meet specific functional requirements. The following sections focus on the effects of scaffold 3D microenvironment, hardness, and morphology on adipogenic differentiation.

#### 3.4.1. 3D Microenvironment of Scaffolds

Compared with traditional 2D cultures, 3D cultures significantly enhance the adipogenic differentiation potential of stem cells, enabling adipogenesis with reduced exposure to adipogenic induction stimuli [85]. This phenomenon indicates that the 3D microenvironment provides a more physiologically relevant context for stem cell differentiation, representing a significant advancement in tissue engineering and regenerative medicine [86]. In 3D cultures, adipocyte-specific genes such as PPARγ, C/EBPα, aP2, and adiponectin are rapidly and strongly induced, demonstrating the significant advantage of 3D cultures in promoting adipogenesis [87]. Moreover, 3D culture systems not only provide robust mechanical support for cells but also stimulate adipogenesis and lipolysis while reducing lactic dehydrogenase (LDH) secretion, indicating superior biological and mechanical performance for ATE [88]. For example, 3D silk fibroin protein scaffolds significantly affect the proliferation and spreading of rat bone marrow cells. In adipogenic environments, these scaffolds up-regulate the expression of adipogenesis-related genes (e.g., PPARγ2, lipoprotein lipase, aP2), highlighting the critical role of scaffold material properties in regulating gene expression profiles associated with adipogenic differentiation [89].

#### 3.4.2. Hardness and Morphology of Scaffolds

Scaffold hardness is a critical determinant of stem cell differentiation. Studies have shown that hBMSCs maintain high viability on both soft (0.5 kPa) and stiff (23.5 kPa) scaffolds. However, hBMSCs exhibit a greater tendency to spread on soft scaffolds, accompanied by a stronger adipogenic differentiation tendency. This differentiation is associated with reduced levels of caveolin-1 (CAV1, a marker of caveolar structure), increased levels of Yes-associated protein (YAP), and decreased YAP phosphorylation. As a transcriptional coactivator, YAP enhances the expression of adipogenesis-related genes. Notably, down-regulation of CAV1 expression via small interfering RNA (siRNA) further enhances the adipogenic differentiation of hBMSCs, suggesting that CAV1 suppression may facilitate adipogenesis through activation of the YAP signaling pathway. Therefore, manipulating CAV1 expression represents a promising strategy to direct hBMSCs differentiation towards adipogenesis in ATE [90].

In addition to hardness, scaffold morphology also affects adipogenic differentiation. Researchers compared the in vivo adipogenic potential of fibrous-structured atelocollagen scaffolds and honeycomb atelocollagen scaffolds. Fibrous-structured atelocollagen scaffolds effectively stimulated a robust adipogenic response in vivo, whereas honeycomb atelocollagen scaffolds triggered an acute inflammatory reaction and failed to support regenerative tissue growth within the scaffold matrix. These findings confirm that the fibrous microstructure of atelocollagen scaffolds is a key factor in inducing adipogenic responses, highlighting the critical role of scaffold morphology in determining ATE outcomes [91].

DAT is a unique scaffold material for ATE, which can be extracted from discarded human adipose tissue. The strategy of using DAT as a bioactive matrix and incorporating it into an in situ polymerizable hydrogel facilitates the delivery of ADSCs to effectively fill small or irregular soft tissue defects. In ADSC/scaffold composites containing DAT particles, adipogenic differentiation is significantly enhanced, indicating that DAT particles regulate stem cell differentiation through cell-ECM interactions [92].

In summary, the adipogenic differentiation of adipose tissue, a core process in ATE, is comprehensively regulated by internal regulatory factors, extracellular environmental factors, exogenous substances, and scaffold materials. These factors interact synergistically to determine the efficiency and quality of adipogenic differentiation. With the continuous advancement of scientific research, our understanding of the regulatory mechanisms underlying adipogenic differentiation will be further deepened, providing theoretical support and technical guidance for the optimization of ATE strategies and its clinical application.

## 4. Effect Factors of Vascularization of Adipose Tissue

**Figure 4:**
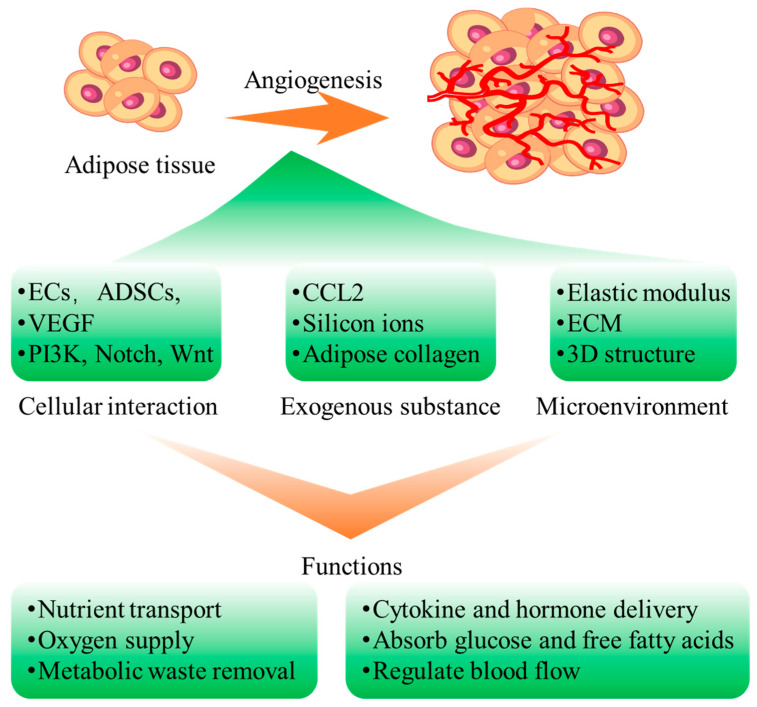
Factors affecting angiogenesis in adipose tissue including cellular interactions, exogenous substances and microenvironment, and the importance of angiogenesis in adipose tissue, such as nutrient transport, oxygen supply, metabolic waste removal, cytokine and hormone delivery, absorb glucose and free fatty acids and regulate blood flow.

Adipose tissue is highly vascularized, with each adipocyte being connected to at least one capillary. The networks of capillaries aids in the spread of adipose tissue metabolites into the bloodstream. Moreover, the role of blood vessels in adipose tissue is especially critical during the growth of adipose tissue and the differentiation of adipocytes. They serve as channels for delivering nutrients and oxygen to the tissues, which are essential for cell activity and the maintenance of tissue functionality [20] (Figure 4). As a highly vascularized tissue, the survival, growth, and functional stability of adipose tissue whether native or engineered are inherently dependent on an intact vascular network. Therefore, clarifying the factors and mechanisms regulating adipose tissue vascularization is a key focus in ATE research, which is crucial for improving the quality and long-term survival rate of engineered adipose tissue in clinical applications.

### 4.1. Importance of Adipose Tissue Vascularization

Vascularization has been recognized as a pivotal factor affecting the quality, volume, and long-term survival of newly formed adipose tissue, whether in physiological development or engineered tissue construction. The process of adipocyte differentiation and proliferation is intimately connected with angiogenesis [93], and the coordinated interplay between adipogenesis and angiogenesis highlights the complex and interdependent nature of tissue development and regeneration in ATE. Specifically, angiogenesis provides the necessary nutrient and oxygen supply for adipocyte growth and differentiation, while adipocytes secrete pro-angiogenic factors to promote vascular network formation, forming a mutually beneficial regulatory loop.

Therefore, the creation of a stable vascular system is critical to fostering the growth and survival of adipose tissue, especially for engineered adipose tissue transplanted in vivo. Vascularization in ATE presents numerous challenges, among which the establishment of stable vascular networks is the core. Only a functional vascular network can ensure the healthy survival of transplanted adipose tissue and avoid complications such as graft necrosis caused by nutrient and oxygen deficiency. To address this issue, researchers are actively exploring a variety of strategies, including the use of growth factors and biomaterials to enhance angiogenesis, with the goal of ensuring that transplanted adipocytes receive a sufficient supply of nutrients and oxygen to facilitate growth, maturation, and differentiation.

The primary biological processes for creating new blood vessels in tissues are vasculogenesis and angiogenesis [94]. Angiogenesis involves the expansion of the existing vascular networks through the activation, proliferation, and migration of endothelial cells, leading to the formation of new vessels and networks. Typically, this process occurs within existing vascular formations, such as capillaries and postcapillary venules [95]. In adult organisms, the formation of new blood vessels is mainly dependent on the angiogenic mechanism. When angiogenesis is impeded, the ability to form new capillaries is compromised, leading to a range of abnormalities within the affected adipose tissue. These abnormalities may manifest as altered lipid storage, impaired adipocyte metabolism, and disrupted adipose tissue homeostasis, and over time, they can progress to more serious consequences such as tissue necrosis [96].

In clinical practice, the failure of blood vessels to adequately grow around transplanted tissues following surgery is a common challenge that can lead to suboptimal outcomes in various surgical procedures, particularly in procedures such as skin transplantation and breast reconstruction [97,98]. By promoting angiogenesis, researchers aim to improve the success rate of surgeries that rely on tissue transplantation or reconstruction, thereby enhancing patient outcomes, reducing complications, and increasing satisfaction with the results of these medical procedures. Thus, the research and development of angiogenic strategies represent a critical area of investigation in the field of regenerative medicine and tissue engineering, with direct implications for advancing ATE clinical applications.

### 4.2. Mechanisms of Adipose Tissue Vascularization

The process of adipose tissue angiogenesis is extremely complex, involving the interactions of different cell types, regulatory factors, and signaling pathways. Neighboring cells in adipose tissue, such as endothelial cells, fibroblasts, lymphocytes, and macrophages, primarily produce and secrete regulatory factors that modulate angiogenesis [99,100]. In addition, exogenous substances and the local microenvironment (especially the microenvironment provided by biomaterial scaffolds) also play essential roles in regulating vascularization (Table 1). The following sections elaborate on the core mechanisms of adipose tissue vascularization from three aspects: cell interactions, exogenous compounds, and the microenvironment of biomaterial scaffolds (Table 1).

#### 4.2.1. Cell Interactions

**Figure 5:**
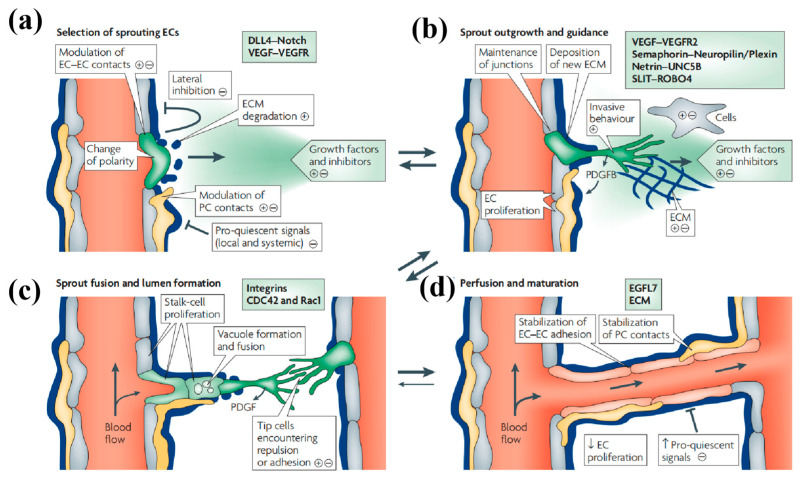
Angiogenic sprouting. (**a**) Sprouting is controlled by the balance between pro-angiogenic signals (+), such as vascular endothelial growth factor (VEGF), and factors that promote quiescence (−), such as tight pericyte (PC; yellow) contact, certain extracellular matrix (ECM) molecules or VEGF inhibitors. In conditions that favor angiogenesis, some endothelial cells (ECs) can sprout (green), whereas others fail to respond (grey). Sprouting requires the flipping of apical–basal polarity, the induction of motile and invasive activity, the modulation of cell–cell contacts and local matrix degradation. (**b**) The growing EC sprout is guided by VEGF gradients. Other signals may include attractive (+) or repulsive (−) matrix cues and guidepost cells in the tissue environment. Release of platelet-derived growth factor B (PDGFB) by the tip cells promotes the recruitment of PCs to new sprouts. EC–EC junctions need to be maintained after lumen formation to prevent excessive leakage. (**c**) Adhesive or repulsive interactions that occur when tip cells encounter each other regulate the fusion of adjacent sprouts and vessels. Lumen formation in stalk ECs involves the fusion of vacuoles but other mechanisms may also contribute. (**d**) Fusion processes at the EC-EC interfaces establish a continuous lumen. Blood flow improves oxygen delivery and thereby reduces pro-angiogenic signals that are hypoxia-induced. Perfusion is also likely to promote maturation processes such as the stabilization of cell junctions, matrix deposition and tight PC attachment. Growth factor withdrawal can trigger sprout retraction and endothelial apoptosis. DLL4, delta-like-4 ligand; EGFL7, epidermal growth factor ligand-7; ROBO4, roundabout homologue-4 (also known as magic roundabout); VEGFR2, VEGF receptor-2. Reproduced with permission from ref. [101]. Copyright 2007 Springer Nature.

Cellular interactions are the core driving force of adipose tissue vascularization, and the coordinated cooperation between various cell types (including endothelial cells, adipocytes, ADSCs, and perivascular cells) and their secreted factors is essential for the formation and maturation of vascular networks. The process of vascular remodeling can be divided into two main stages [101] (Figure 5). During the initial phase, endothelial cells secrete diverse growth factors and post-injury cytokines, which in turn promote the proliferation and migration of surrounding cells. As endothelial cells interact and connect with each other, a new layer of endothelial cells is gradually formed. Subsequently, with the maturation of this newly developed endothelial cell layer, vascular smooth muscle cells initiate the repair process. They alter their shape and arrangement through proliferation and migration to adapt to the evolving vascular architecture, including the branching, merging, lengthening, and restructuring of blood vessels, ultimately forming an extensive and functional vascular network [110,111].

Within adipose tissue, adipocytes and endothelial cells form a mutually regulatory relationship. The adipocytes promote angiogenesis by secreting endothelial cell-specific mitogens and pro-vascular growth factors, while endothelial cells are crucial in angiogenesis [112]. They proliferate, branch, connect, and restructure to create functional vascular networks that supply oxygen and nutrients to adipose tissue [113]. The proliferation of endothelial cells is highly sensitive to PI3K signaling; both excessive and insufficient PI3K signaling can lead to abnormal angiogenesis. The main signaling pathways involved in adipose tissue vascularization include the VEGF signaling pathway [114] and Wnt signaling pathway [115]. The VEGF signaling pathway is activated by VEGF and promotes the proliferation and migration of endothelial cells [116], while the Wnt signaling pathway, which is closely related to the regulation of cell proliferation and differentiation, plays an important role in blood vessel formation and maintenance [117,118]. Understanding the intricate interplay between these signaling pathways is fundamental to elucidating the mechanisms underlying adipose tissue vascularization and its implications for ATE and regenerative medicine.

ADSCs have been demonstrated to significantly enhance both angiogenesis and adipogenesis in ATE, serving as a key regulatory cell type in vascularization. Cell tracking studies have revealed that the newly formed tissues in ATE are predominantly of host origin, indicating that ADSCs facilitate tissue regeneration by recruiting and inducing the differentiation of host cells within the ECM. The combination of ADSCs and ECM creates an inductive microenvironment that supports adipose tissue regeneration, providing insights into the mechanisms of adipogenesis and contributing to a deeper understanding of the complex processes involved in adipose tissue regeneration [75].

Intercellular communication has a synergistic effect on angiogenesis. For example, cultivation of endothelial progenitor cells (EPCs) or HUVECs alone does not effectively promote angiogenesis. This co-culturing HUVECs with ADSCs results in the formation of a stable neovascular system, whereas co-culturing EPCs with ADSCs does not lead to vascular structure formation. When co-implanted with ADSCs, HUVECs surpass EPCs in promoting angiogenesis and in recruiting perivascular cells to stabilize the vasculature in vivo. By differentiating into mature adipocytes, perivascular cells and cellular factories, ADSCs secrete mediators that modulate the local environment to stimulate angiogenesis of de novo adipose tissue and vasculogenesis of co-implanted HUVECs [119]. Moreover, ADSCs from human abdominal adipose tissue co-cultured with HUVEC or EPCs stimulate the neovascularization potential of fibrin constructs on the chorioallantoic membrane of fertilized chicken eggs. Additionally, they directed the formation of capillary-like structures (angiogenesis) in the transplanted human endothelial cells, with the effect of HUVECs being superior to that of EPCs [120].

By subcutaneously injecting ADSCs and human microvascular endothelial cells (HMECs) into severe combined-immunodeficiency mice, it was observed that by day 3, HMECs could form blood vessels within modules containing embedded ADSCs. These vascular structures persisted for up to 90 days. The co-transplantation of ADSCs with HMECs exerted anti-apoptotic and pro-angiogenic effects on HMECs, promoting vascular stability and growth [121]. ADSCs have the capability to transform into adipocytes and endothelial cells, thereby supplying an adequate cell count for vascularized adipose tissues. The clarification of mutual reliance of adipogenesis and angiogenesis, stemming from interactions among endothelial cells, stem cells, and adipocytes at the molecular scale, offers fresh insights into the formation of vascularized adipose tissues [122].

#### 4.2.2. Exogenous Compounds

To enhance adipogenesis and angiogenesis, researchers have investigated various strategies, including the incorporation of exogenous chemokine (C-C motif) ligand 2 (CCL2), silicon ions, and adipose collagen fragment. CCL2 has been shown to enhance angiogenesis and adipogenesis both in vitro and in vivo. In addition, silica expander capsules were found to significantly improve construct stability and increase vessel strength and the number of oil red O-positive lipid droplets, facilitating tissue remodeling and upregulated PPARγ expression [123]. Bioactive silicon ions could enhance the lipogenic differentiation of hBMSCs by stimulating the expression of lipogenic differentiation switches such as PPARγ and CEBPA. In addition, silicon ions could promote angiogenesis and adipogenesis by modulating the interaction between hBMSC-derived adipocytes and HUVECs, and inhibit the dedifferentiation of co-cultured adipocytes. In vivo studies further demonstrated that the designed composite hydrogel (silicon-sodium alginate with cocultured adipocytes and HUVECs) has the ability to release bioactive silicon ions, which can significantly stimulate angiogenesis and adipose tissue regeneration [124].

The adipose collagen fragment containing a variety of adipogenic and angiogenic proteins is capable of releasing multiple adipokines in the acellular adipose matrix. In vitro, higher expression of lipogenic markers (PPARγ, CEBPA) and a greater number of tubular structures were observed in the adipose collagen fragment-treated group. In vivo, adipose collagen fragment incorporated into acellular adipose matrix or decellularized dermal matrix implanted in nude mice could induce highly vascularized mature adipose tissue [125].

#### 4.2.3. Microenvironment of Biomaterial Scaffolds

**Figure 6:**
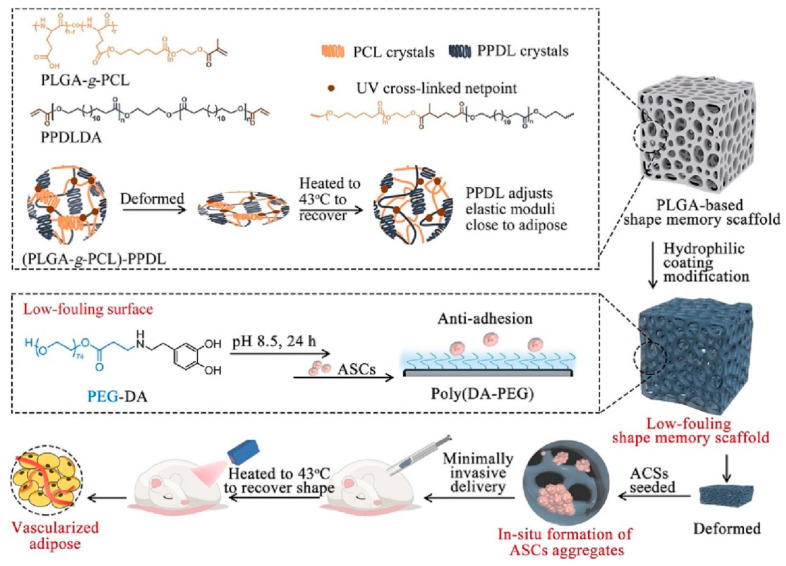
Illustration of a low-fouling shape memory scaffold for in situ formation of ADSCs aggregates and vascularized adipose construction. Reproduced with permission from ref. [127]. Copyright 2023 Elsevier.

Scaffolds provide a critical platform for cell survival and can physically and chemically stimulate blood vessel formation [126]. The scaffold has a unique structure and morphology that mimics the microenvironment of ECM. The surface of the scaffold can be modified with a wide range of bioactive molecules such as growth factors, cytokines, etc. An example of such a scaffold is an innovative low contamination shape memory scaffold fabricated by crosslinking poly(omega-pentandecalactone) with polyglutamic acid-g-Polycaprolactone and further modified with dopamine-modified polyethylene glycol. This scaffold was designed to mimic the elastic modulus of adipose tissue, and the scaffold with coating was designed to prevent adhesion of ADSCs while facilitating their aggregation. In vitro and in vivo experiments showed that this scaffold significantly enhanced the expression of angiogenic and adipogenic factors, thereby facilitating the formation of vascularized adipose tissue [127] (Figure 6). Due to the fact that cells at the inner part of aggregates are naturally exposed to mild hypoxia, this leads to the triggering of hypoxia-inducible factor 1-alpha (HIF-1α) expression to enhance the secretion of VEGF and FGF-2 [128]. The improved adipogenic differentiation is probably due to a significant increase in PPARγ, and the lower cytoskeleton tension of ADSCs in the aggregates [129]. Further, the highly elastic poly-L-lactide-co-epsilon-caprolactone copolymer, combined with human adipose tissue-derived ECM hydrogel, has been used for vascularized ATE. This hydrogel-PLCL scaffold promotes the expression of M2 macrophages, which play an important role in tissue remodeling and regeneration [130]. In addition, PLGA hydrogel scaffolds have a significant swelling hydrophilic network that weakens cell adhesion to the scaffold, while promoting ADSC aggregation to form spheroids. These spheroids upregulate the expression of angiogenic genes (VEGF and FGF-2) by enhancing hypoxia-induced paracrine secretion, and promote the regeneration of vascularized adipose tissue in nude mice [131].

Researchers have developed a protocol denoted as decellularized skin/adipose tissue flap, which consists of ECM and intact vasculature. The decellularized skin/adipose tissue flap retains the advantages of vascular pedicle, microcirculatory vessels and sensory neural networks, while preserving its 3D nanofibrillar structure. When ADSCs and HUVECs are combined with the flaps, they are able to form 3D aggregates and vascular-like structures in vitro. Re-implantation of 3D aggregated flaps into nude mice results in angiogenesis and tissue remodeling, with predominant M2 macrophage infiltration and significant adipose tissue formation at 3 months post operation. These results suggest that decellularized skin/adipose tissue flap co-cultured with ADSCs and HUVECs is a promising platform for vascularized soft tissue flap engineering [132].

The vascularization of adipose tissue plays a crucial role in ATE effectiveness and is vital for addressing obesity, metabolic disorders, and formulating soft tissue repair approaches. The vascularization of adipose tissue still faces significant challenges despite research improvements, which is a key impediment to its widespread application. To improve the clinical use of ATE, researchers need to investigate this issue further.

## 5. ATE Biomaterial Scaffolds

In the field of tissue engineering, scaffold materials play a pivotal role in providing a 3D microenvironment for the growth and differentiation of stem cells for tissues repair and regeneration. Thus, they have a profound effect on the growth status of cells and the ultimate functions of tissues. Scaffold materials can be classified into different types based on their structures and properties, each with unique advantages and limitations. The selection and design of these materials are critical to achieving effective tissue repair and regeneration [16].

### 5.1. Hydrogels

Hydrogels are a class of soft biomaterial composed of a 3D network of hydrophilic polymers that can simulate the ECM [133,134,135,136,137]. The ECM, synthesized and secreted by animal cells into the extracellular space, is a macromolecular substance distributed on the cell surface or between cells [138]. It primarily consists of polysaccharides, proteins, and proteoglycans, forming a complex network structure. The ECM not only supports and connects tissue structures but also participates in regulating cellular physiological activities and tissue formation [139,140]. Its components encompass structural proteins (such as collagen and elastin), specialized proteins (e.g., fibronectin), and proteoglycans. The ECM finds extensive applications in biomedical fields, including regenerative medicine and tissue engineering, where it serves as a natural material capable of inducing tissue repair and regeneration [141]. The advantages of ECM materials lie in their biocompatibility and ability to mimic the in vivo extracellular matrix environment, fostering cell adhesion, proliferation, and differentiation while mitigating immune rejection responses [142,143]. Hydrophilic polymer networks can be formed into hydrogel networks through certain chemical or physical crosslinking [144,145].

Recent advances in this field have made the hydrogel stand out in tissue engineering due to their unique characteristics not found in other biomaterials, including superior biocompatibility, biodegradability, flexibility, ductility, and self-healing features, etc. [146,147,148]. A comparative study by Molina et al. further confirmed that hydrogels exhibit better cytocompatibility and cell adhesion capacity compared to traditional porous polymer scaffolds in adipose tissue engineering (ATE), which is attributed to their water-rich 3D network structure that closely mimics the native ECM of adipose tissue [149]. The 3D structure of hydrogels can provide support for cells, while their high water content is conducive to cell migration, the exchange of nutrients and metabolites, and the promotion of cell growth, proliferation, and angiogenesis [150,151,152,153]. For example, ADSCs can be uniformly distributed and stably cultured, enriched and differentiated in gelatin–alginate hydrogel. Cell differentiation in the hydrogel could be further regulated by the addition of bFGF [154]. It was found that poly(N-isopropylacrylamide) hydrogels combined with DAT powder and grafted with bioengineered mussel adhesive protein exhibited better volume maintenance, neovascularization, and adipogenesis effects [155].

The therapeutic efficacy of current clinical strategies is unsatisfactory due to the high demand for personalization and timely vascularization in the adipose regeneration process. In one study, researchers fabricated composite hydrogel scaffolds containing gelatin methacrylate anhydride and calcium silicate bioceramic hydrogel scaffolds to promote vascularized adipose tissue restoration. In vitro experiments demonstrated that the scaffold not only offered customized structural control but also markedly enhanced the adhesion, proliferation, migration, and differentiation of HUVECs and 3T3-L1 preadipocytes. In vivo, the scaffold assisted in the restoration of adipose tissue vascularization beneath the skin of nude mice [156].

Adipose tissue contains basement membrane proteins and growth factors, and studies have shown that the hydrogel composed of these substances was able to better promote the differentiation of 3T3-L1 preadipocytes compared to tissue culture dishes and Matrigel. Furthermore, when the hydrogel was transplanted around the vertebral root bundles in the upper abdomen of implanted rats, significant fat formation was observed and the fat content was significantly higher than that of Matrigel [157]. Matrigel is a basement membrane matrix extracted from the Engelbreth–Holm–Swarm mouse sarcoma. Its primary components include laminin, type IV collagen, entactin, and heparan sulfate proteoglycan, among others [158]. It also contains various growth factors and enzymes [159]. This matrix mimics the natural microenvironment of ECM, providing abundant nutrients and growth factors to support cells. It is widely applied in cell culture, organoid culture, drug screening, and other fields [160]. The advantages of Matrigel lie in its nutrient richness and ability to mimic in vivo microenvironments, helping cells maintaining normal physiological functions and morphology.

However, its complex composition and significant batch-to-batch variability may compromise the reproducibility and stability of experimental results. Additionally, its potential immunogenicity restricts its use in certain research applications. Moreover, the relatively high price of Matrigel increases experimental costs. This study provides an environment to study cell–matrix interactions in adipogenesis, which could lead to new materials for ATE. In addition, hydrogels prepared with DAT and small intestinal submucosa (SIS) or aortic adventitia extravascular matrix (Adv) can enhance adipogenic differentiation of ADSCs by activating PPARγ and C/EBPα, recruit macrophage populations and promote M2-type macrophage polarization [161].

Hydrogels have broad potential applications in ATE. By further optimizing the composition and structure of hydrogels, their biocompatibility and degradation properties can be matched to the growth cycle of biological tissues, making them more suitable for clinical applications. In addition, when combined with advanced technologies such as 3D and 4D printing, scaffolds with specific structures and functions can be tailored to better meet the needs of different tissue repairs [162].

### 5.2. Porous Materials

The development of porous biomaterials for ATE centers on their capacity to recapitulate the structure and function of native adipose tissue. Featuring a highly porous architecture with pore size, distribution, and connectivity comparable to those of the ECM, these materials create a favorable three-dimensional microenvironment for cell adhesion, proliferation, differentiation, and neovascularization [163,164]. Their distinctive porous microstructure provides cells with an in vivo-like growth niche that supports nutrient and oxygen exchange, ECM deposition, cell–cell interactions, and subsequent tissue formation [165,166].

Compared to hydrogels, porous biomaterials exhibit a fully interconnected 3D porous network that offers ample space for cell growth and migration, facilitating the formation of complex tissue-like structures [167]. Pore size and distribution can be precisely tailored to accommodate diverse cell and tissue requirements. Furthermore, their superior mechanical strength and stability enable them to resist external mechanical loads, a key advantage for load-bearing applications in tissue engineering [168].

Ideal porous biomaterials should have good biocompatibility to avoid a host immune responses, as well as an adjustable biodegradation rate to ensure gradual degradation as new tissue is formed, thereby avoiding long-term residual effects on the body. For example, aliphatic polyester materials including porous biomaterials based on PLA, PGA and PLGA copolymers are widely used in ATE due to their good biocompatibility and controllable degradation properties [169,170,171]. Researchers developed a method based on a network of fused porous DAT microcarriers to create microporous bead foams. Due to the pores and channels that formed between individual microcarriers, there was a secondary level of porosity within the bead foams that could be systematically altered by adjusting the size, shape, and packing density of the beads. The method could be easily modified to create foams made from different collagen sources, such as ECM from different tissues, and unique molds could be used to specify the general scaffold geometry. ADSCs demonstrated significantly higher levels of glycerol-3-phosphate dehydrogenase activity, because culturing ADSCs in softer matrices that more closely resemble natural soft tissue promotes a rounder cell shape and improves the process of adipogenic differentiation [172].

For the treatment of soft tissue abnormalities, adipose tissue engineering is a viable substitute for the current surgical methods. However, finding the right scaffold that not only provides a good habitat for cells but also enables the creation of tailored tissue constructs is challenging, especially in breast surgery. Wiggenhauser, P.S., et al. prepared sponge-like polyurethane scaffolds by mould casting using formaldehyde as a blowing agent, and polycaprolactone scaffolds by mold deposition modelling [173]. Polyurethane scaffolds have an interconnected spherical pore network with adjustable porosity, pore size and biodegradability. Polycaprolactone scaffolds have a highly regular stacked fiber structure and a fully interconnected microporous network. Both scaffolds were inoculated with human adipose-derived progenitor cells, cultured using femoral arteriovenous vascular rings and implanted in nude mice for angiogenesis. Moreover, cells adhered to both scaffolds and differentiated into adipocytes. In vivo, angiogenesis and adipose tissue formation were observed in both constructs after 2 and 4 weeks. This is mainly attributed to the fact that polyurethane scaffolds have a network of highly regular stacked-fiber architecture pores, which are interconnected by small and randomly distributed openings. This structure contributes to the accumulation of cells inside the scaffold, forming high-density cell aggregates. Intercellular interactions and locally high cell densities in cell aggregates are important factors in promoting adipose differentiation. Polycaprolactone scaffolds have a highly regular stacked fiber structure that forms a fully interconnected microporous network, an open structure that facilitates cell invasion. However, fibrous tissue formation and adipogenesis were more pronounced on the polyurethane foam scaffolds than on the polycaprolactone prototype scaffolds. Due to the expanding gas during manufacturing, the polyurethane foam displayed a more haphazard network of spherical pores. The pore network is more twisted because the connections between the cavities are smaller in size. This resulted in aggregates with locally elevated cell densities [173,174].

The biocompatibility and degradation properties of porous materials can be optimized by fine-tuning parameters such as their chemical composition, pore structure, surface properties, and crosslinking degree. This enables the personalized design of scaffold structures and functions to enhance their ability to support cell growth, differentiation, and angiogenesis [175]. Specifically, by tailoring pore size, connectivity, surface hydrophilicity and the grafting of bioactive molecules, high performance scaffolds can be designed to promote cell adhesion, proliferation and differentiation, as well as biodegradation to match the rate of tissue regeneration, thereby advancing the development and innovation of ATE.

### 5.3. Microspheres

Microspheres, defined as microscopic particles composed of various materials including polymers, are widely used in biomedical applications. These particles, typically ranging in size from nanometers to micrometers, exhibit unique properties such as high surface area-to-volume ratio and controlled drug release capabilities. In fat tissue engineering, microspheres serve as scaffolds, carriers, or drug delivery systems, offering tailored microenvironments that support cell growth, differentiation, and vascularization. Through carefully designed microsphere platforms, researchers can precisely regulate cell behaviors during the tissue formation process, significantly improving the long-term survival and functionality of fat grafts [176].

As injectable biomaterials, microspheres exhibit significant potential as carriers for targeted cell delivery, specifically in the context of transporting stem cells to adipose tissue that are damaged or in need of repair. This approach harnesses the inherent properties of microspheres, including their ability to encapsulate and protect cells during transport, as well as their capacity for controlled release at the site of interest. Consequently, microspheres facilitate the directed homing of stem cells to adipose tissue lesions, fostering their differentiation into adipocytes and promoting the regeneration of functional adipose tissue. By encapsulating cells within microspheres, the cells can be protected from the external environment during transplantation. Moreover, the degradation properties of the microspheres can enhance cell adhesion, proliferation, differentiation and control the rate of cell release at the target site.

Microspheres can serve as fundamental building blocks for the construction of ATE scaffolds. By adjusting the dimensions and morphologies of these microspheres, it becomes feasible to mimic the native microstructure of adipose tissue, thereby offering an optimal interface that promotes cell adhesion and proliferation. This strategy not only enhances the structural fidelity of the engineered tissue but also fosters a favorable microenvironment conducive to cellular growth and differentiation, ultimately facilitating the creation of functional adipose tissue constructs [177]. To clarify whether collagen’s bioactivity or the space the mesh provides can promote adipogenesis, 3T3-L1 murine preadipocytes were successfully aggregated into cell clusters utilizing biodegradable, porous microspheres approximately 50 μm in diameter, which served as effective, buoyant, and cell-adhesive microcarriers. In contrast to non-porous microspheres, the formation of spherical aggregates reaching up to 1000 μm in size was readily achieved, encapsulating a greater number of viable cells. This finding underscores the importance of microsphere porosity in enhancing cell–microsphere interactions and promoting the assembly of preadipocytes into functional aggregates, which are crucial for ATE applications [178].

The 3D network of porous microspheres can promote cell–cell interactions and tissue integration. For example, one study used sodium alginate/gelatin composite-based microspheres, which provided adipocytes with a growth environment similar to that of natural fat lobules, demonstrating their great potential as tissue engineering scaffolds [179]. Another study combined the use of PLGA microspheres with ADSCs to successfully achieve cell differentiation into adipocytes in an animal model, validating the potential applications of this strategy in non-invasive soft tissue repair [180].

In addition, the microspheres can be coated with biologically active molecules, such as angiogenic factors, to effectively promote adipocyte differentiation and neovascularization—a key step in ensuring the healthy survival of the new adipose tissue. The gelatin-based microspheres exhibited a two-phase bioreaction strategy by regulating the concentration of gelatin. First, neovascularization is induced by the rapid release of angiogenic factors (e.g., bFGF), followed by the controlled release of insulin and insulin-like growth factor I (IGF-I) to promote preadipocyte proliferation and differentiation. The flexibility of this method lies in the ability to regulate the rate of protein release according to gelatin concentration. Microspheres made with low concentrations of gelatin release the drug more rapidly, making them suitable for the quick delivery of bFGF. In contrast, microspheres with high concentrations of gelatin release protein more slowly, which is appropriate for the prolonged delivery of insulin and IGF-I. This offers a novel means for the precise regulation of protein release in tissue engineering applications [81]. Moya, M.L., et al. evaluated alginate microsphere-delivered fibroblast growth factor (FGF-1) in the ATE vascular pedicle model to promote type I collagen neovascularization and adipogenesis [181]. The results showed that FGF-1 microspheres stimulated the formation of vascular networks in an in vitro 3D co-culture model more effectively than bolus administration of FGF-1. In in vivo experiments, implantation of FGF-1 microbeads into a chamber surrounding the exposed femoral pedicle of a rat resulted in a significant increase in vascular density, with more than 48% of vessels with associated mural cells at 1 and 6 weeks compared to FGF-1 injection. Despite increased angiogenesis, adipogenesis was less than 3%. This suggests that FGF-1 delivered by alginate microbeads induces more durable neovascularization in vitro and in vivo but does not significantly promote adipogenesis. Further studies are needed to better understand how local environmental factors help promote adipogenesis [181].

Despite these observed inconsistencies, such as FGF-1 microspheres significantly promoting angiogenesis but not directly inducing adipogenesis, these results highlight the intricate nature of biological responses and the need for further investigation. Nonetheless, microsphere technology, with its distinctive structural attributes and adaptable functional capabilities, has emerged as a critical component in ATE research. Its role in facilitating angiogenesis and cell differentiation must not be overlooked, as it lays the groundwork for more effective and safer clinical strategies in fat regeneration therapies.

### 5.4. Fibrous Materials

Fibrous materials are structured materials consisting of a series of fibrous substances, which may be natural, synthetic, or modified. The structure of fibrous materials contains a large number of fiber-to-fiber and fiber-to-air interfaces, with very loose bonds between fibers, resulting in very high effective porosity. In addition, fibers are substances with a large aspect ratio and fine diameter, exhibiting bending deformation and good shape adaptability. From a microstructural perspective, some fibrous materials are very similar to plant tissues or the ECM. Fibrous materials such as silk [182], PLA [183] and PLCL [184] have been used in medical fields such as dressings, surgical sutures due to their excellent biocompatibility and high toughness [185]. Collagen microfibers could encapsulate mature adipocytes to be bioprinted in a gellan gum support bath. These multilayered bioprinted tissues retained high viability even after 7 days in culture [186]. The foreign body reaction in a tissue engineering chamber can be reduced by the effect of electrospun polycaprolactone fibers [187]. Polycaprolactone hollow fiber membranes with uniform morphology and good mechanical and transport properties supported the attachment and proliferation of ADSCs [188].

Silk is a natural protein fiber composed of sericin and silk fibroin, which has a wide range of applications in biomedical field. Gobin et al. prepared a composite material using chitosan and silk fibroin as a scaffold to deliver ADSCs for the repair of full-thickness skin injury in mice. The scaffolds loaded with ADSCs not only promoted wound closure but also increased local microvessel density compared to the scaffolds alone and the control group without cell transplants [189]. Moreover, the loaded ADSCs effectively secreted EGF, TGF-β1, and VEGF, which accelerated the repair of skin defects in diabetic rats [189]. Effective surgery is required to address the clinical issue of post-subcutaneous tumor removal. A study proposed a high-performance scaffold for tissue regeneration. Wangkulangkul et al. [190] developed a silk fibroin scaffold with molecular and physical stability and favorable mechanical properties using a salt immersion method. 3T3-L1 cells were seeded on the scaffolds, and it was found that silk fibroin was able to support cell adhesion, spreading, elongation, and aggregation, demonstrating good biological properties. Melt electrowriting, an additive manufacturing method, creates easily manageable fibrous frameworks with precise shapes to facilitate cell infiltration. As a proadipogenic platform for hMSCs, melt electrowriting scaffolds coated with ECM have been developed. Compared to controls, adipogenic differentiation was enhanced on all fiber scaffolds [191].

Fibrous materials have shown great potential in biomedical field, particularly in advancing tissue repair technologies and regenerative medicine. In the future, research will focus on refining the composition and properties of fibrous substances to develop more efficient and safer medical solutions, revitalizing clinical practice and expanding the range of therapeutic options. This process requires advancements in fiber processing technology, as well as the integration of material biocompatibility and functionality, ensuring that scientific precision supports the development of biomedical products to meet growing health needs.

### 5.5. Decellularized ECM Scaffolds

Decellularized extracellular matrix (dECM) scaffolds are derived from native tissues through decellularization processes (e.g., physical, chemical, or enzymatic treatments) that remove cellular components while preserving the native ECM structure, composition, and bioactive molecules [192]. As a “native-like” scaffold, dECM closely mimics the in vivo microenvironment of adipose tissue, retaining key ECM components such as collagen, elastin, proteoglycans, and endogenous growth factors (e.g., VEGF, bFGF), which are crucial for regulating cell adhesion, proliferation, and adipogenic differentiation [193,194].

In ATE, dECM scaffolds are typically derived from adipose tissue itself, as well as other tissues such as small intestinal submucosa (SIS), aortic adventitia, and skin, due to their abundant ECM content and biocompatibility [195]. Adipose-derived dECM (Ad-dECM) is particularly attractive because it retains the specific ECM composition of adipose tissue, including adipocyte-derived growth factors and structural proteins, which can directly promote the adipogenic differentiation of ADSCs and support the formation of functional adipose tissue [196]. For example, a study by Liu et al. reported Ad-dECM scaffolds by decellularizing porcine adipose tissue and found that these scaffolds significantly enhanced the adipogenic differentiation of ADSCs compared to synthetic scaffolds, as evidenced by increased expression of adipogenic markers (PPARγ, C/EBPα) and lipid accumulation [196].

The advantages of dECM scaffolds lie in their excellent biocompatibility, low immunogenicity, and inherent bioactivity, which reduce the risk of immune rejection and promote tissue integration [197]. However, challenges remain, including batch-to-batch variability (similar to Matrigel), difficulty in controlling the degree decellularization, and limited mechanical strength, especially for dECM derived from soft adipose tissue [198]. To overcome these limitations, researchers have combined dECM with synthetic polymers (e.g., PLA, PLGA) or natural polymers (e.g., gelatin, alginate) to enhance mechanical stability while retaining the bioactivity of dECM [199]. For instance, dECM-polycaprolactone composite scaffolds showed improved mechanical properties, promoting the tube formation of endothelial cells, the migration and maturation of vascular smooth muscle cells, and ECM deposition both in vitro and in vivo [200].

With the continuous optimization of decellularization technologies (e.g., mild enzymatic treatment, nanotechnology-assisted decellularization), dECM scaffolds are expected to become a promising candidate for clinical ATE applications, as they can provide a truly native-like microenvironment for adipose tissue regeneration [201].

### 5.6. 3D-Printed/Bioprinted Architected Scaffolds

3D-printed and bioprinted architected scaffolds have emerged as a revolutionary technology in ATE, enabling the precise fabrication of scaffolds with customized geometry, pore structure, and composition that match the specific needs of adipose tissue regeneration [202]. Unlike traditional scaffold fabrication methods (e.g., freeze-drying, salt leaching), 3D printing/bioprinting allows for the control of scaffold parameters (pore size, porosity, connectivity) at the microscale, which is critical for simulating the native adipose tissue structure and promoting cell infiltration, proliferation, and neovascularization [203].

3D-printed scaffolds for ATE are typically fabricated using synthetic polymers (PLA, PLGA, PCL), natural polymers (gelatin, alginate, collagen), or their composites, due to their printability, biocompatibility, and controllable degradation properties [204]. For example, 3D printing was used to fabricate silk fibroin scaffolds with a highly regular porous structure, which provided better soft tissue structural integrity compared to collagen [205]. Similarly, stereolithography 3D printing was employed to fabricate gelatin methacrylate scaffolds with precise pore geometry, which enhanced the proliferation and differentiation of adipose-derived stem cells in situ [206]. Bioprinting, a subset of 3D printing, involves the direct printing of cells (e.g., ADSCs, preadipocytes) together with bioinks (hydrogels, dECM, or polymer composites) to fabricate cell-laden scaffolds, which can directly mimic the native tissue structure and accelerate tissue regeneration [207]. For instance, solubilizing dECMs were bioprinted by macromolecular crowding, and the resulting bioprinted tissues retained high cell viability after 3 days of culture [208]. Another study used bioprinting to fabricate hyaluronic acid-laden polylactic acid scaffolds, which successfully improved cell functionality by increasing the expression of chondrogenic gene markers and specific matrix deposition, thereby promoting tissue formation [209].

The main advantages of 3D-printed/bioprinted scaffolds are their customization capability, precise structural control, and ability to integrate cells and bioactive molecules into the scaffold structure [210]. However, challenges include the limited printability of some bioactive materials, the need for biocompatible and biodegradable bioinks, and the maintenance of cell viability during the printing process [211]. Future research will focus on developing high-performance bioinks, optimizing printing parameters, and combining 3D printing with other technologies (e.g., 4D printing, nanotechnology) to further improve the functionality of architected scaffolds for ATE.

### 5.7. Prevascularized Constructs

Prevascularized constructs are a type of advanced ATE scaffold designed to address the critical challenge of insufficient vascularization in large-volume adipose tissue regeneration, which often leads to cell death and graft failure [212]. These constructs integrate vascular components (e.g., endothelial cells, vascular progenitors, or preformed vascular networks) into the scaffold structure to promote rapid neovascularization and ensure the supply of nutrients and oxygen to the inner layers of the engineered adipose tissue [213].

The fabrication of prevascularized constructs typically involves two main strategies: (1) co-culturing adipogenic cells (ADSCs, preadipocytes) with vascular cells (HUVECs, vascular smooth muscle cells) in a scaffold to induce the formation of de novo vascular networks; and (2) integrating preformed vascular structures (e.g., vascular rings, microvessels) into the scaffold before implantation [213,214]. For example, a study fabricated prevascularized constructs by co-seeding endothelial cells and mesenchymal cells in glycidal methacrylate-hyaluronic acid scaffolds and found that the co-culture system spontaneously formed lumen-like structures and functional endothelial networks in the prevascularized tissues [215]. In another study, pre-differentiated rat adipose-derived stem cells and arteriovenous vascular bundles were cultured on porous hydroxyapatite-polyamide scaffolds, and the resulting constructs showed rapid angiogenesis in vivo [216].

Bioactive molecules (e.g., VEGF, bFGF, angiopoietin-1) are often incorporated into prevascularized constructs to further promote vascularization and adipogenesis [217]. For instance, alginate microspheres loaded with FGF-1 were used to enhance vascular network formation in a prevascularized ATE model, resulting in significantly higher vascular density compared to bolus administration of FGF-1 [218]. Additionally, prevascularized constructs can be combined with 3D bioprinting to fabricate complex vascular networks that mimic the native adipose tissue vasculature [219].

Despite significant progress, prevascularized constructs still face challenges, including the difficulty in fabricating functional and stable vascular networks that can integrate with the host vasculature, and the need to balance vascularization and adipogenesis [220]. However, with the development of advanced fabrication technologies and a deeper understanding of the molecular mechanisms of vascular-adipose crosstalk, prevascularized constructs hold great promise for overcoming the limitations of current ATE strategies and enabling large-volume adipose tissue regeneration [221].

In summary, ATE biomaterial scaffolds have evolved from traditional single-type scaffolds (hydrogels, porous materials, microspheres, fibrous materials) to diverse, advanced constructs (dECM scaffolds, 3D-printed/bioprinted scaffolds, prevascularized constructs), each with unique advantages and limitations (Table 2). The selection of ATE scaffolds should be based on specific clinical needs (e.g., repair volume, injection requirement, mechanical demand) and the biological characteristics of the target tissue. Future research will focus on optimizing scaffold composition and structure, integrating multiple scaffold types (e.g., dECM-based 3D-printed prevascularized constructs), and combining scaffolds with advanced technologies (e.g., gene therapy, nanotechnology) to further improve the efficacy of adipose tissue regeneration and promote the clinical translation of ATE strategies.

## 6. Model Construction and Evaluation

**Figure 7:**
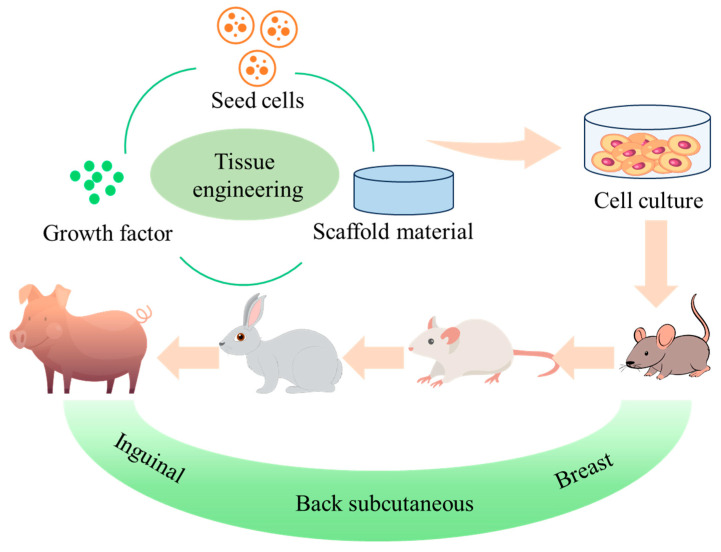
The key elements of ATE include seed cell, growth factor and scaffold material. From in vitro models to in vivo models, including mice, rats, rabbits and pigs.

The construction and evaluation of tissue engineering models involves some strategies, including the selection of appropriate cell types, the design of scaffold materials, the integration of growth factors, and the establishment of in vitro experimental models to simulate the physiological and pathological conditions of target tissues. Through biocompatibility and biodegradability tests, assessment of functional and mechanical properties, and combination with in vitro and in vivo experiments, researchers can systematically construct and evaluate tissue engineering models to provide a scientific basis for clinical applications [222] (Figure 7).

### 6.1. In Vitro Models

In vitro studies provide a rapid, efficient and relatively inexpensive platform for evaluating the biocompatibility and biodegradability of scaffold materials. This research approach not only helps to rapidly screen and optimize tissue engineering scaffolds, but can also be used to gain a deeper understanding of the mechanisms of clinical diseases, to discover new drugs and to evaluate the safety and efficacy of drugs. Moreover, the in vitro simulation of angiogenesis is a powerful tool to study this complex physiological process, allowing the rapid identification of gene networks and signaling pathways involved in angiogenesis. Common in vitro model construction methods include cell-material complexes, organoid culture [223], biological 3D printing [224], and microfluidic chip system [225].

In particular, one study provided empirical evidence for the construction of a tissue engineering model with potential for drug screening. Mouse pluripotent embryonic stem cells were implanted into a polycaprolactone scaffold and induced to differentiate into adipocytes. Microstructural analysis confirmed the similarity of cell morphology, and molecular-level detection (Akt phosphorylation and cyclic AMP synthesis) verified the success of differentiation [226]. Ma et al. comprehensively assessed the proliferative and differentiation capacities of ADSCs, and demonstrated the important role of platelet-rich fibrin (PRF) in promoting ADSC proliferation, endothelial differentiation, and angiogenesis, specifically by activating the ERK and Akt pathways and upregulating the expression of VEGF and IGF-1 [227]. These findings not only provide a theoretical basis for stem cell-mediated vascularization therapeutic strategies but also highlight the potential application of PRF as a biomaterial in tissue engineering.

Traditional in vitro models are usually established using animal-derived serum, such as fetal bovine serum (FBS), which is associated with risks of contamination, compositional inconsistency, and adverse effects on cells. To address these issues, a serum-free restricted co-culture medium was developed and applied to adipocyte/endothelial cell co-culture models. Drawing on the endothelial cell basal medium, this medium incorporates specific growth factors, hormones, and vitamins essential for these cells. It not only serves as an invaluable tool for culturing and investigating vascularized adipose tissue models but also provides a more precise and controlled experimental environment for tissue engineering vascularization studies [228].

Blood vessel formation results from the proliferation and migration of endothelial cells. Therefore, the proliferation status of endothelial cells is an important indicator for the assessment of angiogenesis [229]. Assays for cell proliferation include cell viability, live-dead staining and flow cytometry. Proliferating cells migrate to the target area to form blood vessels, a process that usually involves the secretion of matrix metalloproteinases. Therefore, the migration and proliferation of endothelial cells can be indirectly assessed by measuring MMP levels [230,231]. In addition, the scratch method, a commonly used method to study cell migration and angiogenesis, involves creating scratches in the endothelial cell layer using pipette tips, needles or cell scrapers, followed by observation of cell migration to the scratched area and monolayer cell regrowth [232].

**Figure 8:**
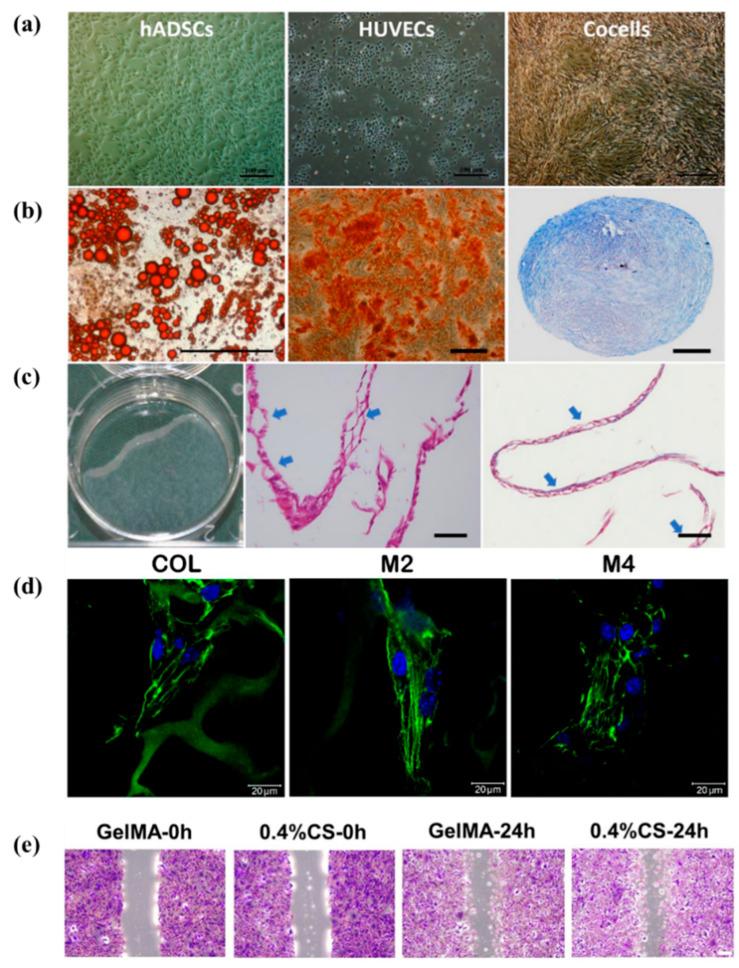
Evaluation methods for adipogenic differentiation and angiogenesis in adipose tissue engineering. (**a**) Morphology of ADSCs, HUVECs and co-cultured cells; scale bar: 100 µm. (**b**) Multiple differentiation induction of ADSCs; Left, Oil Red O-stained adipogenic induced ADSCs, scale bar: 100 µm; middle, alizarin red-stained osteogenic induced ADSCs, scale bar: 100 µm; right, alcian blue-stained chondrogenic induced ADSCs, scale bar: 200 µm. (**c**) left, gross view of the cell sheet co-cultured for 3 days; middle and right, H&E and Masson’s trichrome staining of cell sheet, respectively; blue triangles indicate vacuoles-like structures; scale sheet in a 6-well plate, scale bar: 100 µm. Reproduced with permission from ref. [123]. Copyright 2022 Elsevier. (**d**) Evaluation of undifferentiated ADSCs morphology, adhesion and distribution in the 3D materials by confocal microscopy and fluorescent staining. Reproduced with permission from ref. [79]. Copyright 2020 MDPI. (**e**) Representative images of cell migration with the treatment of pure GelMA and 0.4% CS composite scaffolds for 24 h using the scratch assay. Reproduced with permission from ref. [156]. Copyright 2023 Oxford University Press.

Immunofluorescence staining and statistical analysis can be used to further validate angiogenesis. For example, overexpression of miR-31-5p has been shown to promote proliferation, migration and angiogenesis of endothelial cells [233]. Advances in technology, such as Millicell angiogenesis kit and similar high-throughput tests, have enhanced the efficiency and accuracy of angiogenesis studies. Simultaneously, employing scratch assays and molecular biology methods (e.g., PCR, Western blot) has enhanced our comprehension of the movement, growth, and regulatory processes of endothelial cells [228,229,234] (Figure 8).

Therefore, the development of more efficient induction strategies, such as the use of specific combinations of growth factors, simulation of the microenvironment, precise modulation of physical or chemical factors, and gene editing techniques to enhance differentiation potential, is a key direction for future studies. The construction of optimal culture conditions, including 3D culture systems, the selection of biomaterial scaffolds and the establishment of co-culture systems is key to improving the quality and efficiency of stem cell differentiation. Ensuring the safety of stem cell therapies is critical and requires the assessment of genetic stability during stem cell differentiation, avoiding the risk of teratoma formation, and tracking the stability and functionality of new blood vessels over time. In addition, it is crucial to rigorously regulate and standardize cell origins, preparation techniques and transplantation practices to minimize potential adverse effects.

### 6.2. In Vivo Models

In vivo research is an essential part of tissue engineering, with great implications for the evaluation and refinement of cell therapies, biomaterials, and tissue engineering construction. In vivo studies facilitate the assessment of the biocompatibility, biodegradability, and biological utility of tissue engineering constructs. Scientists can ascertain the safety of biomaterials for clinical use by keeping an eye on factors such as inflammatory reactions, potential toxicity, vascularization, and functional reconstruction, as well as the rate at which the materials biodegrade within the body. Furthermore, complex biological processes can be replicated in studies done on real animals. The extracellular matrix, hemodynamics, and immune responses of animals differ greatly from those of in vitro studies. This allows for the observation of treatment methods’ effects and reactions, as well as the integration of host tissue, differentiation, cell proliferation, and migration, under physiological settings. Moreover, animal models are an essential resource for researching disease causes, testing novel treatments, and drug screening, because many diseases in animals mimic human symptoms. Above all, preclinical research requires animal experimentation as a vital step. To guarantee their safety and efficacy, novel biomaterials or therapeutic strategies must first undergo extensive validation in animal models. This lowers the risks involved in clinical trials.

#### 6.2.1. Mouse and Rat Models

The mouse and rat are the most widely used animal models in experimental studies due to their low cost, rapid reproductive rate and gene diversity. In the field of vascular biology and tissue engineering, mouse and rat models are particularly useful for revealing the complex processes of angiogenesis and testing the performance of novel biomaterials.

In a study on capillary structure, the researchers used a mouse model and succeeded in tracking neonatal endothelial cells over days to weeks by creating wounds on the palms and soles of mice, and observed the generation and changes in blood vessels around the wounds through in vivo imaging and molecular biology testing to study the maturation and maintenance mechanisms of vascular networks. It was found that capillary expansion is driven by the phenomenon of vessel regression. Neonatal endothelial cells possess the capability to alter their location and are extensively dispersed throughout the evolving vascular plexus, but their position becomes stable during adult vascular development. This discovery holds significant implications for comprehending the formation and dynamic balance of blood vessels [235]. Through mouse in vivo experiments, Yoo et al. [15] obtained important findings regarding the efficacy of alginate-based hydrogel scaffolds in adipose tissue engineering. The researchers prepared hydrogel scaffolds by homogeneously embedding adipose tissue in alginate polymer networks and transplanted them into subcutaneous pockets on the dorsal region of mice, with adipose tissue alone as a control. The mouse experiment results showed that the hydrogel exhibited uniform, controllable morphology and good cytocompatibility; compared with the control group, it significantly reduced the inflammatory response after transplantation, improved the survival rate of adipose tissue, and upregulated the expression of adipogenic markers (aP2, lipocalin, and leptin), indicating a higher adipogenic rate. These results, derived from mouse experiments, confirm that the alginate hydrogel scaffold maintains and enhances human adipose tissue adipogenesis by providing protection, promoting nutrient and oxygen exchange, and maintaining structural stability.

Rat involvement is crucial in studies of tissue engineering and vascularization. Their large size, well-developed blood vessels, and ease of care make them a good candidate for a variety of vascularization assessment methods. Dong et al. [236] designed a composite hydrogel of DAT and tremella polysaccharide for culturing ADSCs, which was able to increase cell viability, promote cell proliferation, and improve adipogenic differentiation of ADSCs. To further validate the practical application value of this hydrogel, the researchers conducted a dedicated rat wound healing experiment. By loading insulin into the hydrogel and applying it to rat wound models, the secretion of HGF and VEGF from ADSCs was significantly enhanced. This effect directly contributed to the proliferation, differentiation, and paracrine secretion of ADSCs. Importantly, the rat wound healing study revealed that the collagen content, number, and density of neovascularization at the wound site were significantly increased, which ultimately improved the healing rate. This rat-based experiment not only confirmed the effectiveness of the composite hydrogel but also provided vital in vivo evidence for its potential clinical application. Song et al. [237] prepared a chitosan/β-glycerophosphate/collagen hybrid hydrogel that provided ADSCs with a favorable 3D environment. Through in vivo experiments on rats, the team obtained critical results. The hydrogel demonstrated excellent biocompatibility, effectively enhanced the proliferative ability of ADSCs, and promoted their adipogenic differentiation potential. Moreover, after implanting the hydrogel–ADSC complex into rats, the implanted hydrogel underwent effective absorption and biodegradation in the rat body, and this process was accompanied by the formation of new blood vessels in rat fibroblasts. Genipin was used to crosslink the decellularized adipose tissue in the small intestinal submucosa with the extravascular matrix of the aortic adventitia to generate the composite hydrogels. A rat dorsal subcutaneous implant model was used to study the adipogenic and angiogenic properties of composite hydrogels. The results demonstrated the apparent pro-adipogenic and angiogenic properties of the composite hydrogels, offering a feasible cell-free tissue engineering biomaterial with a wide range of clinical applications. This is mostly caused by the pro-adipogenic qualities of decellularized adipose tissue and the extravascular matrix of the aortic adventitia or small intestinal submucosa [161].

Compared to mice, rats are capable of accommodating bigger grafts, a benefit for assessing implant biocompatibility and their functional integration in tissue engineering. Furthermore, the metabolic rate and fat distribution pattern of rats more closely resemble human characteristics, which makes the experimental results more relevant and reliable by better simulating the physiological state of human adipose tissue. The applications of rat models in tissue engineering and regenerative medicine have demonstrated their unique value in evaluating novel biomaterials, revealing the potential pro-adipogenic and angiogenesis characteristics of materials.

Delving deeply into these tissue engineering and regenerative medicine studies with mouse and rat models has unquestionably enriched the field with invaluable knowledge and understanding. Mouse and rat models stand out due to their distinct benefits, making them ideal for confirming biomaterial effectiveness, assessing cell activity, and evaluating the safety and success of new treatments. These studies not only simulate the human tissue environment and verify the physical, chemical and biological properties of biomaterials, but also provide insight into the interaction mechanisms between biomaterials and living tissues.

#### 6.2.2. Rabbit

Rabbits are widely used as experimental animals in biomedical studies, particularly as bioreactors for antibody production, where they exhibit significant advantages. Their phylogenetic proximity to humans, moderate size, docile disposition, and ease of breeding and maintenance in laboratory settings make rabbits ideal models for studying various human diseases, such as atherosclerosis. Their advantages in terms of life cycle, gestation period, fertility, cost-effectiveness, and availability of genomic and proteomic resources position rabbits as a bridge between smaller rodents like mice and rats, and larger animals like dogs, pigs, and monkeys. Rabbits play a crucial role in translational research activities, including preclinical drug testing and the development of diagnostic methods for patients. The fundamental principle behind using rabbits as experimental models lies in their ability to serve as alternatives when facing difficulties in accomplishing certain research objectives, such as translational research [238].

Recently, with the development of biotechnology, especially innovation in the field of tissue engineering, the applications of rabbit models have been further expanded. Researchers have improved the tissue engineering chamber strategy by combining bioresorbable polycaprolactone chambers with decellularized adipose tissue (DAT). A miniaturized porous polycaprolactone chamber was fabricated, taking into account the size differences between human and rabbit chests, and DAT loaded with bFGF was successfully prepared. In rabbit models, highly vascularized adipose tissue was generated de novo from 0.5 mL of bFGF-loaded DAT, nearly filling a 5 mL polycaprolactone chamber. The newly formed tissue exhibited significantly higher expression of adipogenic genes compared to endogenous adipose tissue [239]. A study using rabbits and tissue engineering chambers demonstrated the spontaneous generation of adipose flaps over a 12-week period, with the tissue showing maturity and stability when transferred, offering a promising approach for breast reconstruction with reduced donor-site complications [240]. Furthermore, to determine a new ATE procedure, researchers relied on rabbit models to conduct key in vivo experiments. Two pre-shaped tissue engineering chambers (round and heart-shaped, 8 mL volume) were bilaterally implanted subcutaneously in the dorsal region of rabbits. The rabbit experiment yielded critical findings, at 8 weeks, the chambered adipose flaps had expanded, and flap volume further increased after chamber removal, with adipose tissue regeneration observed. Between 12 and 24 weeks, non-chambered flaps stabilized in growth and maintained the original chamber shape, while continuously chambered flaps showed no further growth. These results, derived entirely from rabbit experiments, clarified that chamber removal stimulates additional flap growth with good shape maintenance [241].

Adipose tissue not only exists in soft tissue areas, but also in the spine, where the volume of adipose tissues is relatively small but still plays an important role that cannot be ignored. In spinal surgery, laminectomy is commonly used to treat spinal cord compression, but postoperative fibrosis may lead to re-compression of the spinal cord or nerve roots, causing failed back syndrome. To address this issue, researchers have explored methods for epidural fat reconstruction. A study using 36 New Zealand rabbits found that using an ECM hydrogel and ADSCs complex after laminectomy successfully reconstructed epidural fat and reduced the expression of fibrosis and inflammation markers TGF-β and IL-6 in the early postoperative stage, providing a new therapeutic strategy to reduce postoperative complications [242].

The rabbit model has shown great potential in ATE and translational medicine research, providing strong support for the development of new treatment methods and laying a solid foundation for future clinical applications.

#### 6.2.3. Pig

Porcine models play an indispensable role in ATE research due to their highly similar physiological structure to humans. To overcome the challenges of scalability and clinical feasibility in ATE, an innovative strategy has emerged, which is to delay fat injection into customized, additive biomanufactured scaffolds. Researchers conducted a study using immunocompetent minipigs, where they evaluated the effects of prevascularization and subsequent fat injection into medical-grade polycaprolactone scaffolds. After a 24-week observation period, angiogenesis and adipose tissue regeneration were evident across all constructs, with the prevascularization and lipoaspirate group exhibiting the highest relative area of adipose tissue, closely resembling native breast tissue. This study underscores the potential of combining prevascularization techniques with delayed fat injections for large-scale adipose tissue regeneration [243]. Large animal models (e.g., pigs) can overcome the limitations of autologous tissue in breast reconstruction, such as insufficient donor tissue and low surgical success rates with high donor-site morbidity [244]. Researchers have developed a comprehensive pig model to bridge the gap in clinically relevant animal models for breast tissue engineering. This model has achieved remarkable success in generating therapeutically relevant quantities of soft tissue, verified by a 12-month trial involving 60 bioresorbable scaffolds implanted in pigs. The flexibility of this model allows for exploration of various treatment modalities, including fat grafting and augmentation with platelet-rich plasma, further emphasizing its potential as a test platform for scaffold-guided tissue engineering concepts [245].

In conclusion, it is critical to adhere to ethical standards in the design and conduct of animal studies, while minimizing the number of animals used and their suffering. Simultaneously, it is essential to carefully record and analyze experimental results to evaluate the biological characteristics of scaffold materials, such as in vivo safety, immune response, degradability, and potential role in ATE. Additionally, it is necessary to combine animal model results with clinical application requirements to assess their potential efficacy and safety in humans. This process may involve interdisciplinary collaboration among biomedical engineers, biologists, clinicians, and ethicists to enhance the scientific value and clinical applicability of research in the tissue engineering field.

## 7. Applications

**Figure 9:**
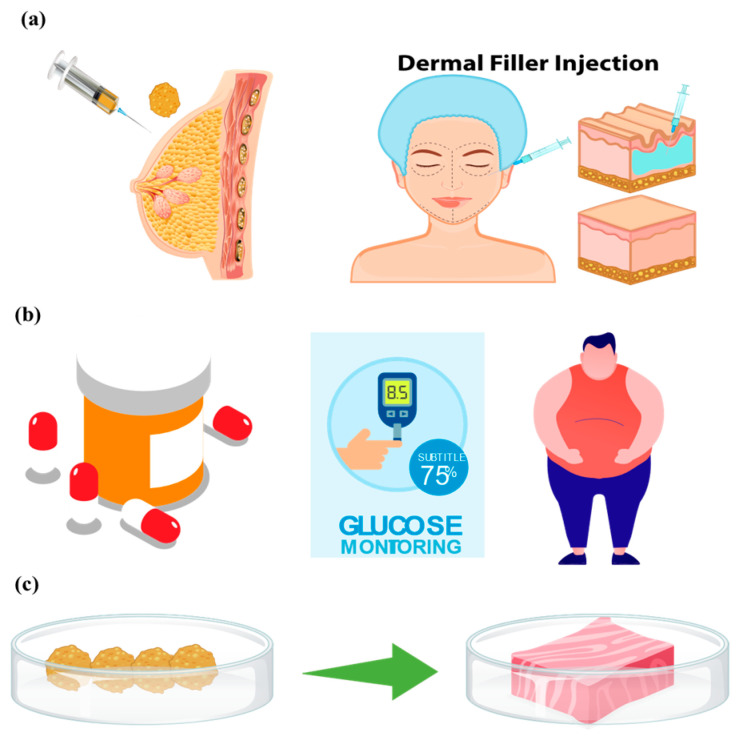
Application of adipose tissue engineering. (**a**) Soft tissue repair and reconstruction. (**b**) Drug screening and disease modeling. (**c**) Produce the volume of large-scale cultured meat.

ATE, as a core interdisciplinary technology integrating biotechnology and engineering, has shown broad application prospects in improving human health, optimizing food supply, and enhancing quality of life. Its specific applications mainly focus on three key directions: soft tissue repair and reconstruction, drug screening and disease modeling, and cultured meat manufacturing (Figure 9). While significant progress has been made in each direction, the clinical translation and large-scale application of ATE still face multiple challenges, including immunogenicity, standardization of adipose-derived stem cells (ADSCs), regulatory barriers, and lack of clinical trial data. This section elaborates on the latest research progress, practical application attempts, and key challenges of ATE in each application direction to provide a comprehensive perspective for its future development.

### 7.1. Soft Tissue Repair and Reconstruction

**Figure 10:**
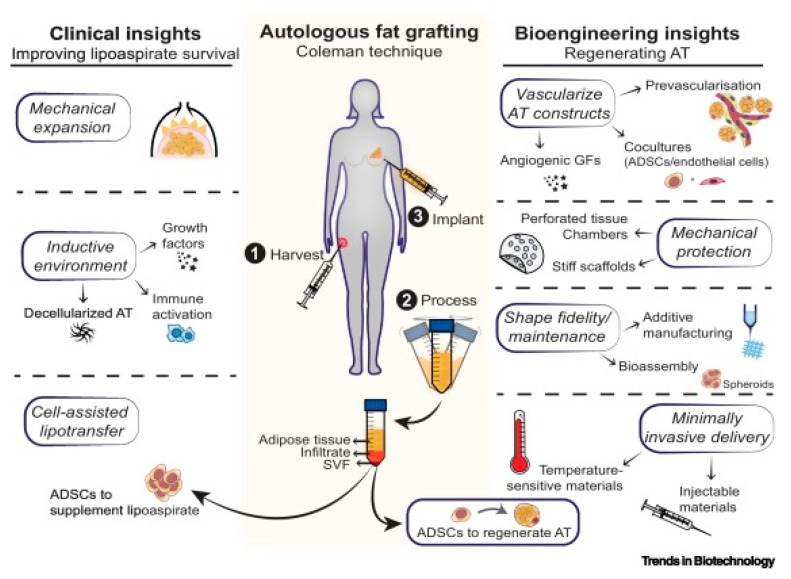
Clinical and bioengineering research concepts to improve and substitute autologous fat grafting. Reproduced with permission from ref. [246]. Copyright 2022 Elsevier.

Soft tissue repair has always been a difficult problem in clinical medicine, and traditional repair methods, such as fat grafting have achieved certain results, but there are problems like damage to the donor area, absorption of the grafted fat, and low survival rate [246] (Figure 10). ATE has emerged as a promising alternative to overcome these limitations, as it enables the construction of engineered adipose tissue that mimics the structure and function of natural adipose tissue, providing new solutions for in vivo and ex vivo repair of soft tissue defects [247].

In clinical research and preliminary applications, regenerative strategies based on ADSCs have been tentatively used in the treatment of Crohn’s disease and wound healing, where ADSCs can preferentially differentiate into adipocytes and help maintain the volume of mature fat grafts [248]. ATE shows particular potential in breast reconstruction, approximately 40% of breast cancer patients require mastectomy, and current post-mastectomy reconstruction methods are often limited by donor site complications and insufficient autologous tissue supply. Autologous tissue-based reconstruction is clinically preferred but constrained by surgical morbidity and donor tissue scarcity. ATE provides a viable solution by constructing replacement adipose tissue, with the key prerequisite of achieving effective vascularization of large-volume tissue constructs [249].

For example, Findlay, M.W., et al. designed an experimental method to implant a superficial circumflex iliac pedicle-based fat flap into the inguinal subcutis, evaluating its efficacy in soft tissue regeneration [244]. Through magnetic resonance imaging and histomorphometric analysis, the researchers systematically assessed tissue growth, angiogenesis, and adipose volume maintenance. The results showed that all implanted chambers were filled with new tissue and formed a complete vascular network within 6 weeks. The initial fat volume in the flap chambers increased significantly at 6, 12, and 22 weeks, indicating effective adipose tissue regeneration. More importantly, the adipose tissue volume was stably maintained for up to 22 weeks after chamber removal, demonstrating excellent durability and potential for clinical application [244].

However, the clinical translation of ATE in soft tissue repair still faces significant challenges. First, immunogenicity remains a critical barrier: engineered adipose tissue constructs, especially those using allogeneic or induced pluripotent stem cells (iPSCs), may trigger host immune responses, leading to graft rejection and failure. Although autologous ADSCs can reduce immunogenicity, their isolation and expansion processes may induce phenotypic changes, increasing immune rejection risks. Second, standardization of ADSCs is lacking: there is no unified standard for ADSC isolation, culture, and differentiation protocols, leading to inconsistencies in cell quality and functional performance between different laboratories and clinical centers, which hinders the reproducibility of ATE constructs. Third, regulatory issues pose obstacles: most ATE-based soft tissue repair strategies are still in the preclinical or early clinical trial stage, and regulatory authorities (e.g., FDA, EMA) have not yet established clear guidelines for the approval of ATE products, including standards for cell sources, scaffold materials, and long-term safety evaluation.

Other existing challenges include optimizing scaffold materials to improve biocompatibility, controllable biodegradability, and bionic structure; expanding cell source diversity to address cell shortage and personalized medical needs; exploring immune tolerance mechanisms and developing immunomodulatory strategies to reduce postoperative rejection; and enhancing the vascularization capacity of engineered tissue to ensure adequate oxygen and nutrient supply. Future research should focus on integrating multidisciplinary technologies (e.g., gene editing, bioprinting, microenvironment simulation) to fine-tune cell behavior and tissue formation, promoting ATE towards more natural and efficient soft tissue repair.

### 7.2. Drug Screening and Disease Modeling

Obesity and its associated metabolic diseases (e.g., type 2 diabetes, hypertension, cardiovascular disease, and certain cancers) have become a global public health burden [250]. Current treatments rely mainly on lifestyle interventions, drugs, and surgery [251], but the development of new weight-loss drugs is a complex, time-consuming, and costly process with an average R&D cost of $2870 million per approved compound and a success rate of only ~10% from Phase I to approval [252,253]. ATE provides a new research platform for obesity and metabolic disease research, enabling the construction of adipose tissue models that closely mimic in vivo physiological conditions, which can be used for drug screening, the study of adipocyte differentiation and metabolism, and exploration of brown adipocyte energy consumption mechanisms [254,255].

Recent research progress has demonstrated the potential of ATE models in drug screening. 3D subcutaneous tissue models constructed using ADSCs can simulate the in vivo adipose tissue microenvironment, enabling efficacy and safety testing of weight-loss drugs while reducing reliance on animal experiments, which is more economical and addresses ethical concerns [256]. For example, researchers have cultured specific ADSC subpopulations in 3D spheroids, stimulated adipogenesis with the pro-inflammatory agent lipopolysaccharide, and found that ADSC spheroids can replicate the unique physiological characteristics of subcutaneous adipose tissue. The deep subcutaneous adipose tissue-derived spheroids showed the highest lipolytic capacity, superficial subcutaneous adipose tissue-derived spheroids had the strongest adipogenic induction, and superficial retinacula cutis-derived spheroids had greater secretory capacity than deep subcutaneous ones. These models can authentically simulate healthy and diseased adipose tissue states, providing a powerful tool for studying cellular and molecular mechanisms and evaluating drug responses [19].

**Figure 11:**
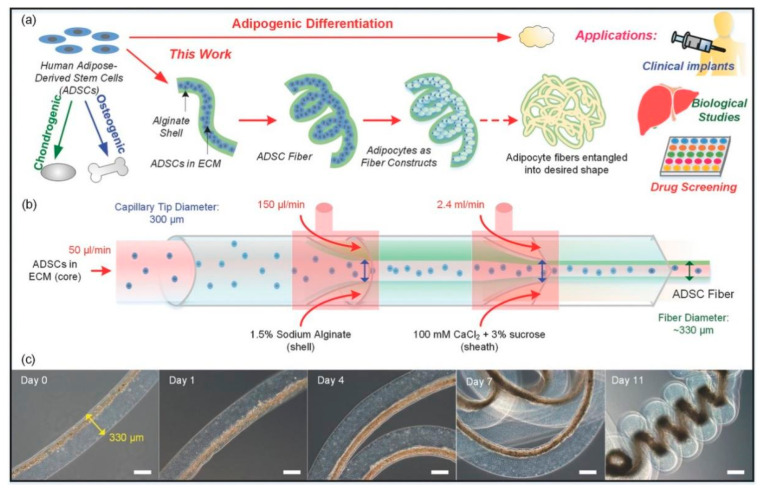
(**a**) Human ADSCs were encapsulated within hydrogel core–shell microfibers, allowed to form into fiber constructs, differentiated to adipocytes, and molded into desired shapes. Potential applications of these adipocyte fibers include clinical implants, biolo­gical studies, and drug screening. (**b**) A schematic drawing of the microfluidic double co-axial device used to fabricate the hydrogel core–shell microfibers. (**c**) Images of the fabricated core–shell microfibers containing ADSCs (not induced for differentiation) on day 0, 1, 4, 7, and 11. Scale bars: 200 μm. Reproduced with permission from ref. [260]. Copyright 2016 Springer Nature.

Brown adipose tissue (BAT), which dissipates energy through heat generation rather than triglyceride storage, has become a key target for obesity prevention and treatment [257]. ATE-based models have been developed to study BAT function. The brown-fat-in-microstrands generated by directed differentiation of pluripotent stem cells can be used to explore BAT energy expenditure mechanisms and its role in metabolic diseases [258]. Additionally, CRISPR-activated (CRISPRa) technology has been used to construct safe and effective ATE constructs with enhanced expression of mitochondrial uncoupling protein 1 (UCP1), a key marker of brown and beige adipocytes. Implantation of these constructs into the inguinal fat pad of mice demonstrated the potential of combining ATE and gene editing to improve cell therapy for obesity, providing a non-immunogenic approach for adipocyte modification and transplantation [259]. Because of their easily observable, controllable, and expandable characteristics, adipocytes are perfect for high-throughput drug screening and testing, biological research, and clinical applications [260] (Figure 11).

Despite these advances, ATE-based drug screening and disease modeling still face notable challenges. First, current ATE models cannot fully replicate the complex in vivo microenvironment (e.g., interactions with immune cells, neural regulation, and systemic metabolic signals), leading to potential discrepancies between in vitro drug screening results and in vivo efficacy. Second, inconsistent ADSC isolation and culture protocols affect the reproducibility of ATE models, making it difficult to compare drug screening results across different studies. Third, ATE models used for drug screening need to meet strict regulatory standards for validity and reliability, but current guidelines for validating such models are still incomplete, hindering their widespread adoption in pharmaceutical R&D.

The clinical relevance of these models also needs to be further confirmed, as the correlation between in vitro drug responses and clinical outcomes remains unclear. To further improve the fidelity of ATE-based drug screening models, it is necessary to address the aforementioned limitation of incomplete in vivo microenvironment simulation. The role of macrophages in adipose tissue inflammation and adipogenesis should be considered: mild inflammation can increase inducible nitric oxide synthase (iNOS) expression, recruit macrophages, and subsequently influence adipogenesis and angiogenesis, highlighting the necessity of integrating immune components into these models [261,262].

### 7.3. Meat Manufacturing

ATE production of cultured meat is a current cutting-edge research area in biotechnology that aims to produce cultured meat with similar taste and nutritional value to real meat by mimicking the natural growth process of living organisms using in vitro cultivation techniques. This technology has significant potential to alleviate global meat supply pressure, reduce the environmental impact of animal agriculture, and provide a more sustainable and healthy meat supply [263].

Research in this field has focused on optimizing raw materials and culture systems to improve production efficiency and product quality. For food safety and cost-effectiveness, biocompatible, food-safe, and low-cost materials are preferred for scaffold preparation. For example, hydrogels prepared from a mixture of carrageenan and konjac glucomannan (plant- and algae-derived polysaccharides) can effectively support cell proliferation and form tissue-like cell spheres. Both porcine subcutaneous pre-adipocytes and fibro-adipogenic precursors cultured in these hydrogels developed mature adipocyte characteristics and higher lipid accumulation, outperforming traditional monolayer cultures and alginate-based 3D cultures. This provides a feasible strategy for the production of “snowflake pork” (marbled pork) [264].

With the advancement of gene editing and stem cell technology, researchers have begun to optimize the adipose content and distribution of cultured meat by editing key genes or inducing stem cell differentiation into adipocytes, making cultured meat closer to conventional meat in taste and texture [265]. Additionally, in-depth studies on cell metabolism and signaling mechanisms during cultivation have helped improve the production efficiency and quality of cultured meat [266].

**Figure 12:**
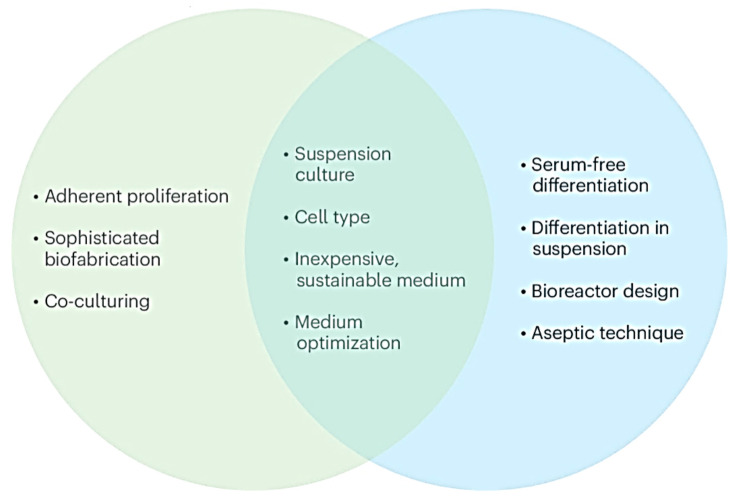
Relevance of current research directions found in primary cultivated meat literature informed by cultivated meat techno-economic analysis. The Venn diagram compares current cultivated meat research topics with important cultivated meat research topics according to techno-economic analysis. The rightmost section therefore lists missing or used understudied research topics. Reproduced with permission from ref. [267]. Copyright 2024 Springer Nature.

However, ATE-based cultured meat production faces the most significant challenges among all application directions, including technical, regulatory, and market-related barriers. First, the selection of suitable seed cells (e.g., stem cells, precursor cells) remains a key issue, as seed cell quality directly affects cultured meat yield and quality (Figure 12) [267]. Currently, there is no unified standard for seed cell isolation, expansion, and differentiation, leading to inconsistencies in product quality. Second, the high cost of cell culture media, scaffold materials, and bioreactors makes cultured meat unaffordable for most consumers; reducing production costs is a prerequisite for large-scale commercialization [268]. Third, regulatory and market acceptance: most countries have not yet established clear regulatory frameworks for cultured meat, including classification (as food or biological product), safety evaluation standards, and labeling requirements. Public skepticism about the safety and “naturalness” of cultured meat also hinders market acceptance.

Other technical challenges include simulating the complex in vivo microenvironment to promote efficient adipocyte growth and differentiation; ensuring cultured meat meets consumer expectations in taste, nutrition, and texture; and optimizing culture conditions (e.g., temperature, humidity, nutrient composition) to enhance adipocyte proliferation and differentiation. Breakthroughs in cost reduction, scale-up technology, and regulatory approval are needed to promote its commercialization.

In summary, ATE has shown great potential in soft tissue repair and reconstruction, drug screening and disease modeling, and cultured meat manufacturing, but its widespread application is hindered by consistent challenges including immunogenicity, ADSC standardization, regulatory barriers, and technical limitations. The readiness level of ATE methods varies by application direction, indicating that most technologies are still in the preclinical or early clinical/pilot stage. Future research should focus on addressing these core challenges through interdisciplinary collaboration, optimizing technology systems, and strengthening clinical and pilot-scale validation. Additionally, close cooperation with regulatory authorities is needed to establish clear guidelines, promoting the safe and effective translation of ATE technologies into clinical practice and industrial applications.

## 8. Conclusions and Outlook

This review systematically synthesizes the core advances and critical bottlenecks of ATE, a field that has evolved from basic cell and material research to preclinical exploration with substantial translational potential. The central conclusion drawn from existing studies is that ATE has achieved remarkable progress in three foundational pillars: seed cell optimization, scaffold material innovation, and vascularization strategy advancement. Specifically, the identification and application of adipose-derived stem cells (ADSCs) and mesenchymal stem cells (MSCs) have resolved the core issue of seed cell availability, while the development of diverse scaffold materials (hydrogels, 3D-printed constructs, prevascularized scaffolds, etc.) and precise regulation of growth factors have laid the foundation for constructing functional adipose tissue that mimics native physiology. Additionally, ATE has expanded its application boundaries beyond clinical soft tissue repair (e.g., breast reconstruction, trauma repair) to drug screening, metabolic disease modeling, and cultured meat production, demonstrating its interdisciplinary value.

However, critical limitations persist that hinder the translation of ATE from preclinical research to clinical practice and industrial scale-up, limitations that have often been overlooked in descriptive summaries. First, the lack of standardized isolation, purification, and culture protocols for ADSCs leads to inconsistent cell quality and adipogenic potential, creating barriers to reproducibility across studies and clinical applications. Second, vascularization remains a bottleneck. While prevascularized constructs and growth factor delivery systems have improved graft survival, the formed vascular networks often lack long-term stability and functional maturity, failing to fully mimic the hierarchical vascular structure of native adipose tissue. Third, immunogenicity and biocompatibility issues of synthetic scaffolds, as well as the high cost of bioprinting and large-scale cell culture, restrict their clinical accessibility. Finally, regulatory frameworks for ATE products (especially in clinical and cultured meat fields) are still underdeveloped, further delaying translational progress.

Looking forward, the sustainable development and translational breakthrough of ATE will rely on targeted solutions to the aforementioned challenges. First, interdisciplinary collaboration between cell biology, materials science, and engineering is essential to develop standardized ADSC culture systems and novel scaffold materials with tunable biodegradability, biocompatibility, and vascularization-promoting properties, such as 4D bioprinting technology that enables dynamic adjustment of scaffold structure and nanomaterial-assisted vascularization. Second, deepening the understanding of molecular mechanisms underlying adipogenic differentiation and vascularization regulation, combined with gene editing technology, will facilitate the construction of more functional and stable engineered adipose tissue. Third, strengthening the establishment of large animal models will bridge the preclinical–clinical gap, while promoting the formulation of unified regulatory standards will accelerate the clinical translation of ATE products. Fourth, in emerging fields such as cultured meat, optimizing fat cell culture efficiency and metabolic pathways through biomanufacturing technology will unlock industrial application potential.

**Figure 13:**
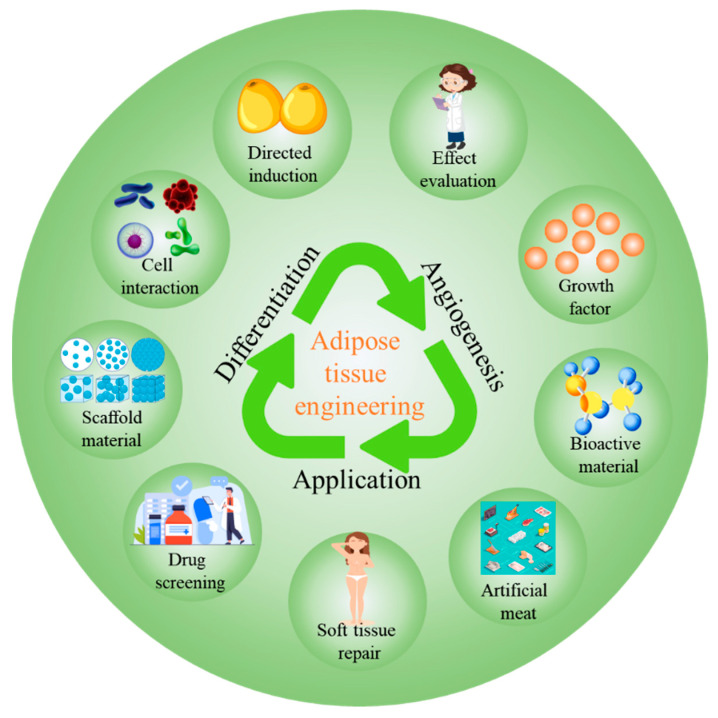
Perspectives for the future development of fabrication strategy and functional applications for adipose tissue engineering.

In summary, ATE holds immense promise for addressing unmet clinical needs in soft tissue repair, metabolic disease treatment, and sustainable food production. Overcoming current technical and regulatory challenges through critical innovation and standardized research will be pivotal to realizing its full potential, ultimately providing comprehensive support for human health from precision medicine to food sustainability (Figure 13).

## Figures and Tables

**Table 1:** Effects of cells and cytokines on angiogenesis.

Cells/Cytokines	Mechanism	References
Lymphocyte	Regulation of angiopoietin 1 and angiopoietin 2 and tyrosine kinase-like receptor 1 and tyrosine kinase-like receptor 2 (Tie1 and Tie2)	[101]
Endothelial cells	Secretion of growth factors Regulation of tip cells and stalk cells	[102]
Fibroblast	Promoting tubule formation in HUVECs Regulation of vascular smooth muscle cell proliferation and differentiation	[103]
Macrophage	Activation of the JAK2/STAT3 signaling pathway Regulation of KDR and HOXB2 expression Inhibition of prolyl hydroxylase and activation of HIF1-α	[104,105]
VEGF	VEGF-A, VEGF-B, VEGF-C, VEGF-D, VEGF-E, PlGF Regulates lymph angiogenesis Regulation of Notch, Wnt/β-catenin, Ang1/tie2, PI3K-AKT signaling pathways	[106]
FGF	Synergy with VEGF Activation of the MAPK signaling pathway outside and protein kinase C (PKC)	[107]
PDGF	Activation of MAPK, PI3K/Akt and Src signaling pathways Promoting differentiation of endothelial cells	[108]
TGF-β	Induction of endothelial to mesenchymal transition Transduction of extra-membrane signals into the membrane via the Smad protein pathway	[109]

**Table 2:** Advantages, limitations and key applications of different types of scaffolds in ATE.

Scaffold Type	Advantages	Limitations	Key Applications in ATE
Hydrogels refs. [133,134,135,136,137,138,139,140,141,142,143,144,145,146,147,148,149,150,151,152,153,154,155,156,157,158,159,160,161,162]	Good biocompatibility/biodegradability; facilitates cell growth and angiogenesis	Low mechanical strength; uncontrollable degradation occasionally	ADSC/3T3-L1 culture; vascularized adipose repair; 3D/4D printing customization
Porous materials refs. [163,164,165,166,167,168,169,170,171,172,173,174,175]	High mechanical strength; supports cell migration and ECM formation	Precise fabrication required; poor bioactivity without modification	Breast repair; large-volume adipose regeneration; angiogenesis
Microspheres refs. [176,177,178,179,180,181]	Cell protection; customizable; bioactive molecule coating available	Lack 3D network; limited biocompatibility of some materials	ADSC delivery; angiogenic factor release; non-invasive soft tissue repair
Fibrous materials refs. [182,183,184,185,186,187,188,189,190,191]	Good biocompatibility/toughness; facilitates cell infiltration	Loose fiber bonding; poor bioactivity of some synthetic fibers	Cell bioprinting; foreign body reaction reduction; soft tissue repair
dECM scaffolds refs. [192,193,194,195,196,197,198,199,200,201]	Native-like microenvironment; low immunogenicity; promotes adipogenesis	Batch variability; limited mechanical strength; difficult decellularization control	ADSC adipogenesis; functional adipose formation; clinical translation
3D-Printed/bioprinted architected scaffolds refs. [202,203,204,205,206,207,208,209,210,211]	Customizable; integrates cells/bioactive molecules; accelerates regeneration	Limited bioink printability; cell viability maintenance challenge	Customized adipose repair; cell-laden scaffold fabrication
Prevascularized constructs refs. [212,213,214,215,216,217,218,219,220,221]	Solves vascularization limitation; enhances cell survival and integration	Difficult stable vascular network fabrication; unbalanced vascularization/adipogenesis	Large-volume adipose regeneration; angiogenesis promotion

## Data Availability

No new data were created or analyzed in this study.
